# Global Change Reshapes Biosynthesis of LC‐PUFA in Northern Lake Food Webs

**DOI:** 10.1111/gcb.71070

**Published:** 2026-08-24

**Authors:** Matthias Pilecky, Kristiina Vuorio, Cyril Rigaud, Marco Calderini, Kimmo K. Kahilainen, Timo J. Ruokonen, Martin J. Kainz, Jussi Vesterinen, Sami J. Taipale

**Affiliations:** ^1^ WasserCluster Lunz – Biologische Station Lunz am See Austria; ^2^ Research Lab for Aquatic Ecosystem Research and Health Universität für Weiterbildung Krems Krems Austria; ^3^ Finnish Environment Institute (Syke) Helsinki Finland; ^4^ Department of Biological and Environmental Science University of Jyväskylä Jyväskylä Finland; ^5^ Lammi Biological Station University of Helsinki Lammi Finland; ^6^ Natural Resources Institute Finland (Luke) Helsinki Finland; ^7^ The Association for Water and Environment of Western Uusimaa Lohja Finland

**Keywords:** bioconversion, compound‐specific stable isotopes, deuterium, ecophysiology, eutrophication, polyunsaturated fatty acids, trophic ecology

## Abstract

Global change is altering the physico‐chemical characteristics of northern lakes through warming, shifting nutrient regimes, and increasing dissolved organic carbon (DOC). These changes are reshaping the trophic structure and nutritional quality of freshwater food webs. To investigate how such environmental gradients influence the production and trophic transfer of long‐chain polyunsaturated fatty acids (LC‐PUFA), we quantified LC‐PUFA biosynthesis in 12 boreal lakes along a trophic gradient. Bayesian models were used to relate biosynthesis to phytoplankton biomass and community composition, while compound‐specific stable hydrogen isotopes and *fads2* gene expression were applied to distinguish dietary acquisition from endogenous n‐3 LC‐PUFA synthesis in zooplankton and planktivorous fish. We further extrapolated LC‐PUFA biosynthesis across 643 boreal and subarctic lakes covering a~7°C temperature gradient and a 1–160 μg L^−1^ phosphorus range using long‐term phytoplankton monitoring data. Phytoplankton LC‐PUFA biosynthesis ranged from 1 to 400 ng g C^−1^ h^−1^, of which the edible (< 35 μm) fraction for zooplankton contributed only 45%–74%. Stable isotope and gene‐expression analysis indicated that biosynthesis rates exceeding ~100 ng g C^−1^ h^−1^ in the edible fraction were required to reduce consumer reliance on endogenous bioconversion to below 10%. Across the 643 lakes, LC‐PUFA production exhibited a unimodal relationship with phosphorus, peaking at intermediate nutrient levels; however, only 166 lakes produced sufficient LC‐PUFA to meet inferred consumer requirements. Nutrient enrichment, warming, and browning acted synergistically to restructure phytoplankton community composition and constrain LC‐PUFA availability. Eutrophication and browning promoted LC‐PUFA‐poor taxa, whereas warming altered biomass distribution and reduced LC‐PUFA production within the edible phytoplankton size fraction. Together, these changes intensified a nutritional bottleneck at the base of aquatic food webs. Our findings demonstrate that global change profoundly modifies phytoplankton community structure, LC‐PUFA production, and trophic transfer efficiency, which may generate cascading effects on the functioning and productivity of northern freshwater ecosystems.

## Graphical abstract

Global change reshapes northern lake conditions, reducing edible phytoplankton LC‐PUFA production. Across 643 lakes, warming, eutrophication, and browning limit dietary LC‐PUFA, forcing zooplankton and fish to rely more on endogenous bioconversion. These shifts intensify nutritional bottlenecks and weaken trophic transfer in freshwater food webs.

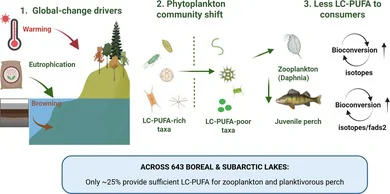

## 1 Introduction

Lakes are highly sensitive sentinels of environmental change, responding rapidly to climate change, nutrient loading, and watershed processes (Adrian et al. 2009; Williamson et al. 2009). Currently, the greatest climate‐related and anthropogenic stressors affecting northern lakes are global warming (temperature increase, reduced ice‐cover time) and intensified lake eutrophication and browning (Kernan et al. 2010; Monteith et al. 2007; Woolway et al. 2020), which can shift phytoplankton community compositions (Rasconi et al. 2017). In addition to providing dietary energy, primary producers supply essential micronutrients (Ruess and Müller‐Navarra 2019), including the omega‐3 long‐chain polyunsaturated fatty acids (LC‐PUFA), particularly eicosapentaenoic acid (EPA) and docosahexaenoic acid (DHA), which are critical for somatic growth, reproductive performance, and eventually survival of aquatic and terrestrial consumers (Brett et al. 2009; Taipale et al. 2018; Twining et al. 2016).

Most consumers are unable to synthesize n‐3 PUFA de novo and therefore depend on dietary supply (Sprecher 2000). However, various animals are capable of converting dietary precursors, such as α‐linolenic acid (ALA), into EPA and DHA through elongation and desaturation steps generally mediated by enzymes encoded by *elovl5* and *fads2* genes (Castro et al. 2016). The capacity for LC‐PUFA biosynthesis varies considerably among consumers due to evolutionary diversification and physiological plasticity of these gene networks (Monroig et al. 2022). Consequently, consumer species with strong endogenous synthesis may be less dependent on dietary EPA and DHA and better adapted to habitats where these compounds are scarce (Twining et al. 2021). These interspecific differences in gene presence and expression create wide variation in conversion capacity, giving consumers with strong endogenous synthesis a competitive advantage when resources are limited. Examples include stickleback populations possessing the *fads2* gene (Ishikawa et al. 2019) and herbivorous Nile tilapia populations thriving in LC‐PUFA‐poor tropical reservoirs through enhanced bioconversion capacity (Magadza et al. 2020; Taipale et al. 2025). Thus, it is proposed that trophic plasticity describes the ability of organisms to flexibly adjust their use of food and energy sources in response to environmental, competitive, or seasonal conditions, thereby altering their trophic niche (resource use) over space or time (Hendry 2016; Sturaro et al. 2021).

Aquatic primary producers differ in their ability to synthesize LC‐PUFA. Diatoms, cryptomonads, chrysophytes, and dinoflagellates are major producers of EPA and DHA, whereas green algae and cyanobacteria primarily synthesize shorter‐chain PUFA (Taipale et al. 2013). Global warming has been predicted to decrease LC‐PUFA production in oceans mainly due to the changes in the homeoviscous adaptation of plankton communities (Hixson and Arts 2016; Holm et al. 2022). In freshwater, ongoing global warming, eutrophication, and browning are expected to alter phytoplankton community composition which may consequently reduce the availability of LC‐PUFA by favoring taxa with limited biosynthetic capacity (Rasconi et al. 2015; Taipale et al. 2016, 2019; Watson et al. 1997). However, recent evidence from a mesocosm experiment suggests that phytoplankton biomass and community composition exert stronger control over LC‐PUFA contents than warming alone (Strandberg et al. 2022). Considering that sub‐arctic and boreal lakes differ in size, depth, color, and other physico‐chemical properties, their responses to ongoing environmental change may vary substantially among each other (Lyche Solheim et al. 2019) with as yet unknown repercussions on LC‐PUFA production (Butcher et al. 2015; Edlund et al. 2022).

Previous studies have reported contrasting responses of zooplankton LC‐PUFA content to eutrophication, lake browning, and warming (Calderini, Pääkkönen, et al. 2023; Keva et al. 2021; Lau et al. 2021; Pilecky, Kämmer, et al. 2022; Strandberg et al. 2023), likely reflecting differences in zooplankton communities, feeding ecology, and biosynthetic LC‐PUFA capacity. Whether freshwater consumers, such as zooplankton or planktivorous fish, can compensate for declining dietary LC‐PUFA through increased endogenous synthesis remains largely unresolved. Such responses can be assessed using *fads2* and *elolvl5* gene expression as indicators of short‐term biosynthetic investment (Rigaud et al. 2023) and compound‐specific ^2^H and ^13^C isotope analyses (CSIA) of longer‐term PUFA conversion (Pilecky et al. 2025; Pilecky, Kämmer, et al. 2022; Pilecky, Mathieu‐Resuge, et al. 2022). Finally, direct quantification of phytoplankton LC‐PUFA biosynthesis is needed to determine whether synthesis rates are sufficient to meet consumer requirements across contrasting lake ecosystems.

The aim of this study was to examine the relationship between LC‐PUFA biosynthesis rates and biomass across different phytoplankton species, and to assess how lake eutrophication, browning, and temperature affect LC‐PUFA biosynthesis by primary producers and its subsequent transfer to aquatic consumers. We first examined the capacity of aquatic consumers to compensate for reduced dietary LC‐PUFA availability by assessing endogenous bioconversion in zooplankton and a common planktivorous fish, juvenile perch ( *Perca fluviatilis* ), across 12 boreal lakes. Consumer reliance on endogenous LC‐PUFA synthesis was evaluated using compound‐specific stable isotope analysis and expression of key biosynthetic genes. We then developed predictive models linking phytoplankton community composition and biomass to LC‐PUFA production rates and applied these models to long‐term monitoring data from 643 Finnish lakes from 1980 to 2024 along temperature and phosphorus gradients. This approach enabled us to estimate lake‐specific LC‐PUFA production and evaluate the extent to which endogenous contribution is required for consumers to meet their physiological LC‐PUFA demands under ongoing environmental change.

## 2 Materials and Methods

### 2.1 Study Sites

**FIGURE 1:**
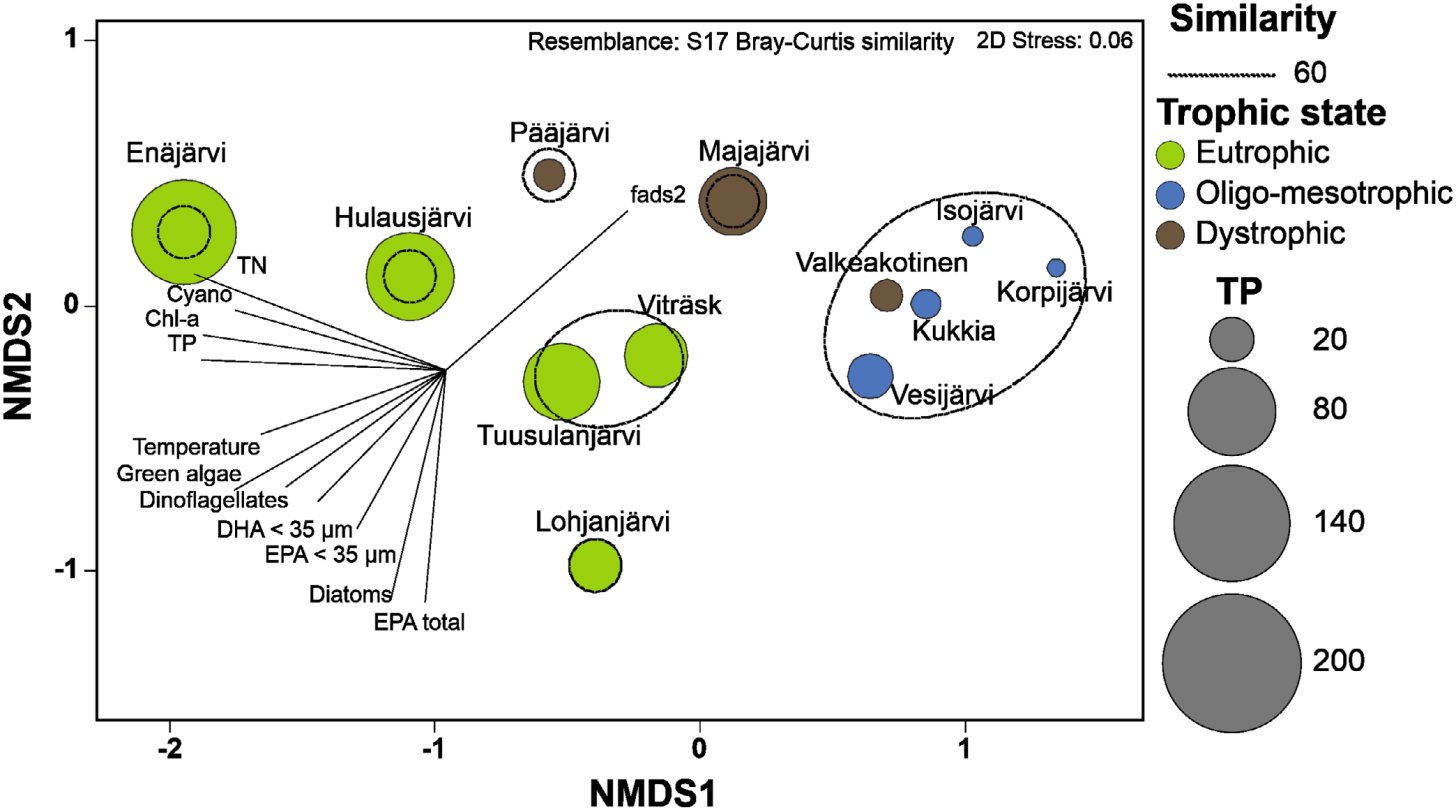
Similarities and dissimilarities among the lakes selected for the field campaign. Lakes were selected to cover a large range of total phosphorus (TP; μg L^−1^; see size of circle) and nitrogen (TN; μg L^−1^) values, resulting in diverse phytoplankton communities and LC‐PUFA biosynthesis rates. Circles cite to the 60% similarities between lakes in CLUSTER analysis.

Twelve boreal lakes spanning a gradient form oligo‐ to eutrophic conditions were selected in central and southern Finland (total phosphorus, TP: 4–140 μg L^−1^; total nitrogen, TN: 413–1814 μg L^−1^; dissolved organic carbon: 5.9–19 mg C L^−1^; Figure 1; Table 1). These lakes were sampled once in July 2021 to assess the LC‐PUFA biosynthesis and to determine stable hydrogen isotope values of PUFA in seston (< 35 μm; i.e., most edible size fraction for herbivorous zooplankton (Burns 1968), zooplankton, and perch ( *Perca fluviatilis* )). Previously published phytoplankton data from these lakes (Calderini, Pääkkönen, et al. 2023) were used to establish relationships between phytoplankton biomass, community composition, and LC‐PUFA biosynthesis. These 12 lakes covered a broad range of phytoplankton assemblages representative of those observed across the larger monitoring dataset (Table S1).

**TABLE 1:** Water chemistry of the study lakes.

	Area [km^2^]	Mean depth‐[m]	Max depth [m]	Chl‐a [mg/L]	P/PO_4_ [μg/L]	N/NH_4_ [μg/L]	N/NO_2_ + NO_3_ [μg/L]	DIN [μg/L]	TN [μg/L]	TN:TP	TP [μg/L]	T [°C]	DOC [mg/L]	Main phytoplankton
Enäjärvi	5	3.5	10	130	3	6	19	25	1814	13	140	19	8.3	Cyanobacteria
Hulausjärvi	2.2	1.1	3.3	80.9	3	11	14	25	1318	13.3	99	18	14.2	Cyanobacteria
Isojärvi	18.3	16.4	69.7	6.5	2	8	46	54	415	83	5	18	8	Golden algae, cyanobacteria
Korpijärvi	31.2	10	41	19.7	2	14	55	69	413	103.3	4	20	6.8	Golden algae
Kukkia	47	5.23	35.6	5.1	2	7	15	22	509	46.3	11	19	8	Cryptomonads, diatoms
Lohjanjärvi	122	12.7	54.5	22.4	2	15	78	93	791	21.4	37	21.7	10.2	Diatoms
Majajärvi	0.034	4	12	26.1	11	12	8	20	1024	17.7	58	18	28.5	Golden algae
Pääjärvi	13.44	15	85	10.7	2	15	1076	1091	1573	121	13	20.1	13.3	Cryptomonads, diatoms
Tuusulanjärvi	6	3.2	10	35.4	5	6	13	19	965	12.7	76	19	10	Cryptomonads
Valkea‐Kotinen	0.041	2.5	6.5	8.1	2	11	5	16	584	44.9	13	19.5	15.3	Rhaphidophytes
Vesijärvi	108	6	40	8.2	3	5	9	14	542	20.8	26	18	6.3	Diatoms
Vikträsk	1.87	4.49	15	40.2	2	6	7	13	644	12.9	50	20.6	11	Cyanobacteria

Abbreviations: DIN, dissolved inorganic nitrogen; DOC, dissolved organic carbon; TN, total nitrogen; TP, total phosphorus.

To assess endogenous LC‐PUFA biosynthesis in consumers, we analyzed *fads2* gene expression in liver tissue and stable hydrogen isotope values in white dorsal muscle tissue of perch (< 15 cm body length), which were predominantly planktivorous (> 75% zooplankton in stomach contents; Table S2) (Calderini, Pääkkönen, et al. 2023; Rigaud et al. 2023). For gene expression analyses, fresh liver tissue samples from each fish were homogenized in DNA/RNA Shield (750 μL) with a TerraLyzer Cell Disruptor and BashingBead (2.0 mm) lysis tubes (Zymo Research, Irvine, CA, USA). For fatty acids analyses, white dorsal muscle tissues were sampled adjacent to the anterior dorsal fin and above the lateral line of each fish. All samples were stored at—80°C prior to further analysis.

### 2.2 RNA Extraction and qPCR

RNA was extracted from liver samples using a Chemagic 360 and the Chemagic Viral DNA/RNA 300 Kit H96 following the manufacturer's instructions (PerkinElmer, Waltham, MA, USA). The RNA was treated with DNAse and its concentration and purity (260–280 nm optical density ratio) were assessed spectrophotometrically (NanoDrop, Thermo Fisher Scientific, Waltham, MA, USA). The 260–280 nm optical density ratio was equal to 1.99 ± 0.12. RNA integrity was assessed for a randomly chosen subset of samples using an Agilent 2200 TapeStation (Agilent Technologies, Santa Clara, CA, USA) following the manufacturer's instructions; all tested samples had a minimum RNA integrity number of 8. The RNA was then reverse transcribed to cDNA using the Maxima First Strand cDNA Synthesis Kit (Thermo Fisher Scientific, Waltham, MA, USA). The effectiveness of the DNase treatment was validated by including randomly chosen negative RT samples. cDNA samples were stored at −20°C.

Primers sequences and parameters were previously reported in Rigaud et al. (2023). In short, the primer sequences for quantitative real‐time PCR analyses (qPCR) for *fads2* and for the two reference genes *ef1‐α* and *β‐actin* were all obtained from the literature (Geay et al. 2016). However, as efficiency values for the qPCR primers were not reported by these authors, we determined them to be equal to 94.4%, 96.9%, and 94.2% for *fads2*, *ef1‐α*, and *β‐actin*, respectively. All amplification reactions were done in a volume of 25 μL containing 2 μL of cDNA, 0.75 μL (each) of the forward and reverse primers (working solution at 10 μM), 9 μL of sterile water and 12.5 μL of 2X Maxima SYBR Green/Fluorescein qPCR Master Mix (Thermo Fisher Scientific). The reactions were run on a CFX96 Real‐Time PCR cycler (Bio‐Rad, Hercules, CA, USA). The protocol was 10 min at 95°C, 40 cycles of 15 s at 95°C, 30 s at 60°C, and 30 s at 72°C, and ended with 10 s at 95°C followed by melting curves from 65°C to 95°C. A single melting temperature peak was observed in the dissociation curves for each pair of primers. No amplification was observed in no‐template controls (sterile water instead of cDNA) and negative RT samples.

For each sample, *fads2* relative gene expression (corrected for efficiency) was calculated according to previous studies of Pfaffl (2001) and Vandesompele et al. (2002). Ideally, reference genes are required to have stable expression levels regardless of biological differences between samples. This can be difficult to achieve when samples are collected outside of a controlled laboratory environment. The stability of reference genes was tested with NormFinder: its algorithm estimates an expression stability score (*S*, considered stable when below 0.5 and very stable when below 0.15) by combining intra‐ and inter‐group variations for each of the reference genes (Ramhøj et al. 2019). The combined stability values for *ef1‐α* and *β‐actin* were *S =* 0.32, which was considered acceptable.

### 2.3 LC‐PUFA Biosynthesis

The biosynthesis rate of LC‐PUFA in the euphotic zone (2× Secchi depth) was assessed using our previously published volumetric production measurements from 12 boreal lakes (Calderini, Kahilainen, et al. 2023). The biosynthesis rate of fatty acids was calculated as the fixation of carbon (Grosse et al. 2015) similarly to what was previously reported for Lake Kariba (Taipale et al. 2025). For that, the excess enrichment of ^13^C in fatty acid planktonic primary producers and dissolved inorganic carbon (DIC) was calculated as follows:

^13^C‐enrichment of fatty acid = AP_Δ_/100 × c_FA_ × C%, where c_FA_ is concentration of fatty acids (μg FA L^−1^), and C% is the relative carbon content of individual fatty acids.

^13^C‐enrichment of DIC = AP_Δ_ = the difference in ^13^C as carbon atom% of DIC after enrichment and at the beginning of incubation.

Biosynthesis rate (ng C mg C^−1^ h^−1^) = (^13^C‐enrichment of fatty acid/^13^C‐enrichment of DIC)/POC/Δt, where POC corresponds to POC concentrations per volume (mg C L^−1^) and Δt is incubation duration, here 2 h. The calculated rate represents hourly fatty acid biosynthesis during the daylight period under boreal summer conditions.

### 2.4 Compound‐Specific Isotope Ratio Mass Spectrometry

Lipid extraction and subsequent fatty acid methylation to form fatty acid methyl esters (FAME) of seston, zooplankton, and juvenile perch samples followed methods as described by (Calderini, Kahilainen, et al. 2023). The analysis of stable hydrogen isotope values of fatty acids (*δ* ^2^H_FA_) was conducted as described previously (Pilecky et al. 2023, 2021), using a Thermo Trace 1310 GC (ThermoFisher), coupled by a ConFlo IV interface (ThermoFisher) to a continuous‐flow isotope‐ratio mass spectrometer (DELTA V Advantage, ThermoFisher). FAME were separated using a VF‐WAXms column (CP9211, 30 m, 0.32 mm ID, film thickness 1 μm; Agilent, Santa Clara, CA). The temperature program started at 80°C, held for 2 min, after which the temperature was ramped at 30°C min −1°C to 175°C, then at 5°C min^−1^ to 240°C, which was held for 35 min. For *δ* ^2^H analysis, FAMEs were pyrolyzed to H_2_ gas by passing them through a high‐temperature graphite conversion reactor at 1420°C.

Samples were run against certified Me‐C20:0 stable isotope reference material (USGS70: *δ* ^2^H = −183.9‰, USGS71: *δ* ^2^H = −4.9‰ and USGS72: *δ* ^2^H = +348.3‰). Weighted‐average combined δ^2^H values of mono‐unsaturated fatty acids (MUFA = C16:1, C18:1) were obtained by integrating over all isoforms (e.g., C18:1n‐7 + C18:1n‐9) because no clear baseline separation could be achieved. FA *δ* ^2^H values ($\delta I_{\mathrm{FA}}$) were corrected for the methyl group addition during methylation according to (Pilecky et al. 2021). Values for *δ* ^2^H were standardized against the Vienna Standard Mean Ocean Water standard (^2^H:^1^H = 155.76 ppm):

δHFA2=H2/HSample1H2/HVSMOW1−1×1000

### 2.5 Phytoplankton Biomass From 643 Boreal Lakes for the Biosynthesis Model

Phytoplankton data were obtained from the Finnish Environment Institute's (Syke) open‐source data portal (HERTTA) and inncluded samples collected during July–August from 1980 to 2024. Lake phytoplankton samples were collected using a water sampler from the upper 0–2 m of the water column, preserved in acidic Lugol's solution, and analyzed using an inverted microscope using the Utermöhl technique with three different total magnifications (Utermöhl 1958, EN‐15204:2007). Phytoplankton were generally identified to the species level and separated into two size groups: small (most edible; < 35 μm) and large (> 35 μm) size fraction based on cell/colony size. Biovolume calculations for each taxon were based on a fixed set of average biovolumes, adjusted for cell size without extensions. If the size variation of a taxon was large, the list included multiple size classes for the taxon. The counting process allows the counting of subcolonies of a certain size or of filament segments 100 μm in length. When estimating the maximum size dimension of each taxon, fine‐grained projections (e.g., spines) and colony mucilage were taken into account, which are not considered when determining cell or colony biovolumes.

In addition to phytoplankton biomass, the database included information on sampling time, depth, and location with numerous physico‐chemical parameters. The dataset encompasses lakes across Finland (60°–69° N), covering broad environmental gradients, including a range of ~7°C annual air temperature, c. 350 mm precipitation, 1–150 μg L^−1^ TP concentrations, and lake areas ranging from 0.01 to 5000 km^2^. In general, temperature, precipitation, and nutrient concentrations decreased from southern to northern Finland. To evaluate how phytoplankton community composition influenced LC‐PUFA biosynthetic rates across contrasting lake ecosystems, lakes were classified according to the Finnish implementation of the EU Water Framework Directive (System B typology). In this categorization, naturally nutrient‐rich lakes (Rr), lime‐rich lakes (Rk), and lakes located in Northern Lapland (PoLa) were first separated from other lakes. The remaining lakes were categorized by size, watercolor, depth, and water retention (Table S2).

### 2.6 Data Analysis

NMDS was used to visualize multivariate patterns in environmental variables, phytoplankton community composition, LC‐PUFA biosynthesis by primary producers, and consumer response variables (LC‐PUFA content of daphnids and perch, and *fads2* planktivorous perch). NMDS was performed in PRIMER 7 using Bray–Curtis similarity on untransformed data. No additional data transformation or standardization was applied. Environmental variables, phytoplankton community composition, and consumer response were related to the ordination using Pearson correlation, implemented as vector fitting to the NMDS configuration.

Further data analysis and graph designing were conducted and plots produced in R (Version 4.4.1, R. Core Team, 2024) using the packages *ggplot2*, *ggpubr*, *rjags*, *mgcv* and *brms*. We used Bayesian regression analysis to quantify how algal group composition predicts EPA and DHA biosynthesis using our experimental data. For each experimental observation *i*, production was modelled with Gaussian likelihoods and linear predictors formed by the inner product of algal group biomass and group‐specific effects with weakly informative priors. Posteriors were propagated to predict LC‐PUFA production of edible and total algal biomass and to model relationships between LC‐PUFA production and consumer isotope/gene‐expression indicators using exponential regression. For better readability of the results, we reported the 95% HDI of our results in Supporting Information (Tables S1–S6).

Zooplankton and fish bioconversion was estimated using Bayesian exponential regression of the relative *fads2* gene expression or isotopic differences (Δ*δ* ^2^H values) between individual LC‐PUFA and ALA. ALA served as a proxy for the isotopic signal of dietary precursor PUFA (Pilecky, Kämmer, et al. 2022; Pilecky, Mathieu‐Resuge, et al. 2022). In contrast to direct PUFA comparisons between consumers and potential diets, this approach accounts for temporal variation in the *δ* ^2^H composition of the dietary resources and provides a more robust estimate of long‐term bioconversion. We estimated the LC‐PUFA production rates at which consumer bioconversion indicators declined to 50% and 10% of their observed maximum values. These estimates are reported as medians with 95% high density interval (HDI) and were subsequently propagated through the lake‐scale modelling framework.

For each lake of the HERTTA database (Table 2), each of the observed algal group biomasses were modelled as a multinomial composition with a conjugate Dirichlet prior. Let $y_{i}=\left(y_{i1},\ldots ,y_{\mathrm{iG}}\right)$ be the total biomass across all samples from lake *i* for G taxonomic groups. We assume

$$y_{i}|p_{i}\sim \text{Multinominal}\left(N,p_{i}\right),\;N_{i}=\sum_{g=1}^{g}y_{\mathrm{ig}}$$

with a weakly symmetric Jeffreys prior on the composition (to avoid zero‐proportion issues) and obtain the posterior by conjugation and calculate the posterior mean for each group.

$$p_{i}\sim \text{Dirichlet}\left(\alpha_{0},\ldots ,\alpha_{0}\right),\;\alpha_{0}=0.5\;y_{i}|p_{i}\sim \text{Dirichlet}\left(y_{i1}+\alpha_{0},\ldots ,y_{\mathrm{iG}}+\alpha_{0}\right)$$

**TABLE 2:** Replication statement.

Study type	Scale of inference	Scale at which the factor of interest is applied	Number of replicates at the appropriate scale
Experimental study	Lakes	Phytoplankton, Seston, Zooplankton and fish samples	12 lakes, each with two total phytoplankton and two seston samples, three zooplankton replicates, and > 5 fish muscle samples
Lake model	Lakes	Algal data (total phytoplankton and edible seston) from lakes	643 lakes with a total of 9225 samples (maximum one value per lake and sampling year)

The dominant group in lake *i* was defined as the group with the largest posterior mean proportion. To quantify uncertainty in dominance, we computed the dominance probability for each group,

Prpig=pihhmaxyi

via Monte Carlo: for each lake we drew 10,000 samples from the Dirichlet posterior and recorded the fraction of draws in which each group attained the largest share.

Lake‐specific LC‐PUFA biosynthesis rates were modelled as smooth functions of environmental parameters (total phosphorus, nitrate, nitrite, ammonia, temperature, color, pH) using generalized additive models. Correlation with conversion/expression data was modelled using an exponential function, and the thresholds were calculated as C_p_ = −log(p)/exponent. Gamma distribution with log was used and smoothing parameters were estimated by REML, which provides stable penalization and guards against overfitting. The model adequacy was verified via qq‐plots and residual versus fitted plots, k‐index and *gam.check* to confirm sufficient *k*, and concurvity to assess non‐linear collinearity. Where residual spatial autocorrelation was suspected, Moran's I was computed on residuals. Uncertainty of smooths is reported as pointwise 95% CIs from the fitted GAM. To locate the optimum (e.g., peak production vs. total phosphorus) and its uncertainty, coefficient vectors from $N\left(\hat{\beta },{\hat{V}}_{\beta }\right)$ were simulated and, for each draw, the smooth over a fine grid was evaluated to obtain the distribution of total phosphorus (median and 95% HDI).

To evaluate how global‐change‐related environmental gradients affected LC‐PUFA biosynthesis, we analyzed all lake‐year observations separately for total phytoplankton and the most edible size fraction (< 35 μm) for zooplankton. Environmental structure among lakes was first explored using principal coordinates analysis (PCoA) based on Gower distances, including LC‐PUFA production and standardized environmental variables such as temperature, total phosphorus, nitrogen availability, water color, pH, chlorophyll‐a, lake area and depth. Direct effects of environmental predictors on LC‐PUFA production were then assessed using generalized additive mixed models (GAMMs) with a Gamma error distribution and log link. Temperature, total phosphorus, nitrogen and water color were modelled as smooth terms, while lake type was included as a categorical predictor and lake identity as a random effect to account for repeated summer measurements. Model robustness was evaluated using reduced‐predictor models and lake‐level bootstrap resampling. Predictor‐only multiple imputation yielded highly consistent model performance across 20 imputed datasets, with adjusted *R* ^2^ ranging from 0.300 to 0.304 and deviance explained from 40.8% to 41.1%. This indicates that model conclusions were robust to missing environmental predictor values.

To estimate how many lakes provided sufficient EPA and DHA for consumers during the sampling period (July), the posterior distributions of modelled lake‐specific LC‐PUFA production rates were combined with the posterior distribution of the critical production threshold derived from our isotope and gene‐expression models. For each MCMC draw, we compared lake‐specific production values to the sampled threshold and counted the proportion of lakes exceeding it. Repeating this across all posterior draws yielded the full posterior distribution of the number of “sufficient” lakes, from which we report the median and 95% HDI.

## 3 Results

### 3.1 Biosynthesis of LC‐PUFA by Phytoplankton of Total and Edible Fraction for Zooplankton in Experimental Lakes

**FIGURE 2:**
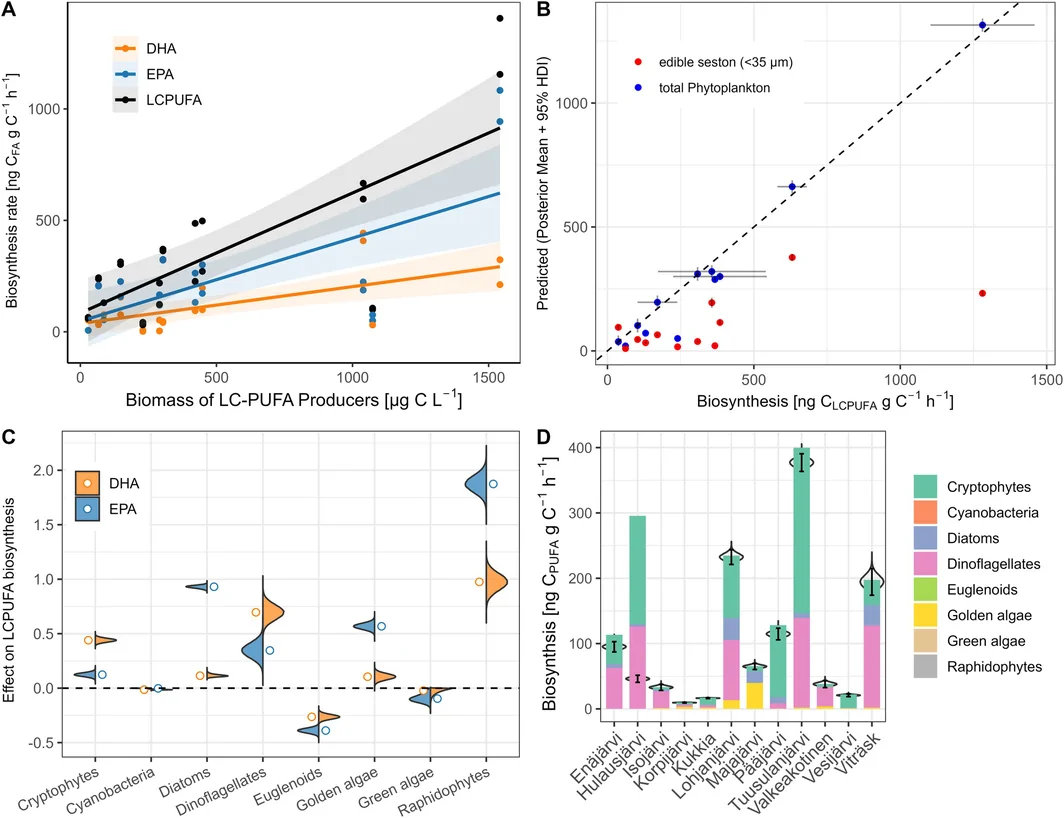
Biosynthesis of DHA and EPA by lake phytoplankton in the experimental setup. (A) Linear regression of the total biomass of LC‐PUFA–producing phytoplankton correlated with the LC‐PUFA biosynthesis rate. (B) A model with Gaussian likelihood and linear predictors quantified the contribution of algal groups to LC‐PUFA biosynthesis and estimated biosynthesis in the edible size fraction (< 35 μm) available to zooplankton. The dashed line indicates a 1:1 relationship for total phytoplankton. (C) Bayesian posterior functions of algal group contribution to biomass normalized LC‐PUFA biosynthesis rates; raphidophytes were the most efficient but are typically too large for zooplankton ingestion. Euglenoids, green algae and cyanobacteria did not produce LC‐PUFA, while increasing the overall biomass. (D) Primary LC‐PUFA biosynthesis of the edible size fraction of the phytoplankton in each lake. Cryptomonads and dinoflagellates contributed most of the LC‐PUFA biosynthesis of the edible size fraction.

The 12 study lakes covered a broad trophic gradient and differed in phytoplankton community composition and LC‐PUFA biosynthesis rates, which ranged from 1.0 to 273 ng g C^−1^ h^−1^. Ordination analysis (NMDS) separated low‐nutrient from eutrophic lake ecosystems characterized by high cyanobacteria biomass (Figure 1). The second ordination axis was positively associated with LC‐PUFA biosynthesis and separated Lake Lohjanjärvi with the highest biosynthesis rates (Figure 1). Expression of the *fads2* gene in planktivorous perch correlated positively with both ordination axes, being highest in low nutrient lakes. The biomass of LC‐PUFA‐synthesizing phytoplankton taxa, including cryptomonads, chrysophytes, diatoms, dinoflagellates, and raphidophytes, was strongly correlated with community‐level LC‐PUFA biosynthesis across the studied lakes (Pearson correlation: *r* = 0.85, *p* < 0.05; Figure 2A). The Bayesian model accounting for taxon‐specific LC‐PUFA biosynthesis efficiency explained nearly all observed variation in LC‐PUFA production (Bayesian *R* ^2^ of 0.97; Figure 2B).

Taxon‐specific model estimates showed clear differences between DHA and EPA biosynthesis (Table S3). DHA biosynthesis was highest in communities containing raphidophytes, dinoflagellates, and cryptomonads, with estimated contributions of 975 ng g C^−1^ h^−1^ for raphidophytes, 696 ng g C^−1^ h^−1^ for dinoflagellates, and 440 ng g C^−1^ h^−1^ for cryptomonads. In contrast, EPA biosynthesis was highest in communities containing raphidophytes, diatoms, and chrysophytes, which accounted for estimated EPA biosynthesis rates of 1880 ng g C^−1^ h^−1^ for raphidophytes, 932 ng g C^−1^ h^−1^ for diatoms, and 568 ng g C^−1^ h^−1^ for chrysophytes. However, dinoflagellates and cryptomonads contributed less to EPA biosynthesis, with estimated rates of 344 and 126 ng g C^−1^ h^−1^, respectively (Figure 2C). Euglenoid biomass was negatively associated with LC‐PUFA biosynthesis, both for DHA (−267 ng g C^−1^ h^−1^) and EPA (−389 ng g C^−1^ h^−1^). Green algae were negatively associated with EPA biosynthesis (−96 ng g C^−1^ h^−1^), whereas cyanobacterial biomass had no detectable effect on LC‐PUFA biosynthesis rates (Figure 2). These negative associations likely reflect the contribution of these taxa to total phytoplankton biomass without corresponding LC‐PUFA production.

Within the zooplankton‐edible phytoplankton size fraction (< 35 μm), the taxonomic basis of LC‐PUFA biosynthesis differed from that of the total phytoplankton community. Cryptomonads and dinoflagellates accounted for most LC‐PUFA biosynthesis, whereas diatoms and chrysophytes contributed relatively little (Figure 2D). Thus, taxa dominating total community biosynthesis did not necessarily contribute proportionally to the edible LC‐PUFA pool available to zooplankton.

### 3.2 Bioconversion Responses in Zooplankton and Fish

**FIGURE 3:**
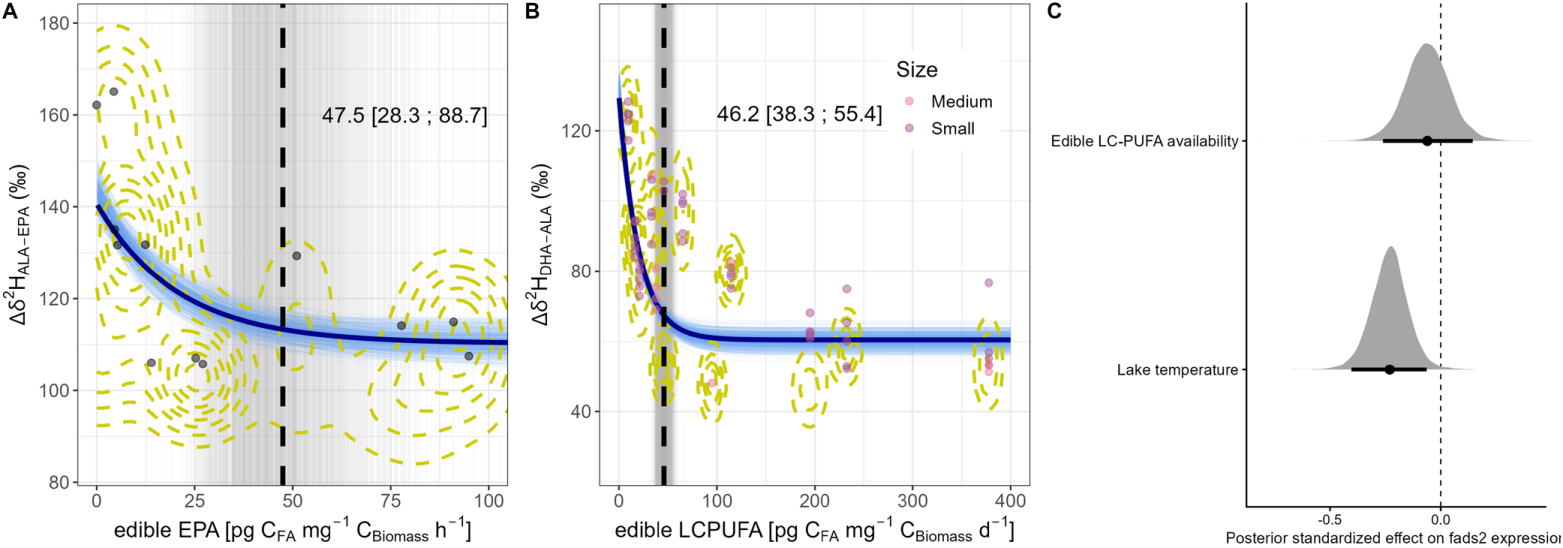
Isotopic and gene‐expression evidence for LC‐PUFA bioconversion by zooplankton and planktivorous perch. Dashed vertical lines in panels A and B indicate the estimated 90% isotope‐response threshold, and yellow dashed lines show the 95% highest‐density intervals of the Bayesian posterior predictions. (A) In herbivorous cladocerans, Δδ^2^H_ALA‐EPA_ was larger at low edible EPA biosynthesis rates and approached a stable offset as edible EPA biosynthesis increased, indicating reduced isotopic evidence for endogenous EPA synthesis at higher basal EPA production. (B) In small‐ and medium‐sized planktivorous perch, Δδ^2^H_ALA‐DHA_ showed a similar saturating pattern in relation to edible LC‐PUFA biosynthesis, indicating reduced reliance on endogenous DHA biosynthesis as basal LC‐PUFA availability increased. Although the response variable is DHA‐specific, LC‐PUFA biosynthesis is shown on the *x*‐axis to represent the integrated basal LC‐PUFA supply linking phytoplankton production to fish. (C) Posterior distributions of standardized effects of log‐transformed bounded edible‐fraction LC‐PUFA availability and lake temperature on perch *fads2* expression. The dashed vertical line indicates zero effect. The temperature effect was consistently negative, whereas the edible‐LC‐PUFA effect was negative in direction but overlapped zero, indicating weaker support for a direct dietary LC‐PUFA effect on fads2 expression after accounting for temperature.

Zooplankton δ^2^H_ALA_ values closely reflected those of seston, particularly for herbivorous cladocerans (Table S4). In contrast, δ^2^H_EPA_ values of the same organisms differed markedly, which correlated with the primary biosynthesis rates of the respective LC‐PUFA. For the zooplankton isotope models, we included only herbivorous cladocerans, whereas predatory cladocerans such as *Leptodora kintii* and copepods (e.g., *Heterocope* sp. and *Limnocalanus* sp.) were excluded. Relative to the primary‐producer baseline, cladoceran Δδ^2^H_EPA_ approached a stable offset of approximately 100‰ at EPA production rates of 46.8 ng g C^−1^ h^−1^, indicating reduced isotopic evidence for endogenous EPA synthesis above this production level (Table S2; Figure 3A). DHA contents in zooplankton were too low in several samples to obtain reliable compound‐specific isotope values; therefore, zooplankton DHA models were not fitted.

Similar isotopic patterns were observed in planktivorous fish muscle. Fish Δδ^2^H_DHA_ approached an offset of approximately 70‰ at primary DHA production rates of 11.5 ng g C^−1^ h^−1^, while the 50% response threshold was estimated at 16.2 ng g C^−1^ h^−1^ (Table S2; Figure 3B). These isotope‐based thresholds were used as the primary basis for estimating the LC‐PUFA production levels at which consumer reliance on endogenous bioconversion declined.

In contrast, *fads2* expression in perch was more strongly associated with lake temperature than with bounded edible‐fraction LC‐PUFA availability (Figure 3C). In the covariate‐controlled Bayesian model, the temperature effect was clearly negative (posterior mean of the slope = −0.231 [−0.404; −0.063]), indicating higher *fads2* expression in colder lakes. The effect of edible‐fraction LC‐PUFA availability was also negative in direction but uncertain (posterior mean of the slope = −0.060 [−0.261; 0.144]). The posterior probability that the absolute temperature effect exceeded the edible‐LC‐PUFA effect was 0.868. We therefore interpreted *fads2* expression as a temperature‐sensitive marker of LC‐PUFA biosynthetic demand, rather than as a quantitative indicator of dietary LC‐PUFA limitation alone.

### 3.3 Predicted LC‐PUFA Biosynthesis Across 643 Finnish Lakes

**FIGURE 4:**
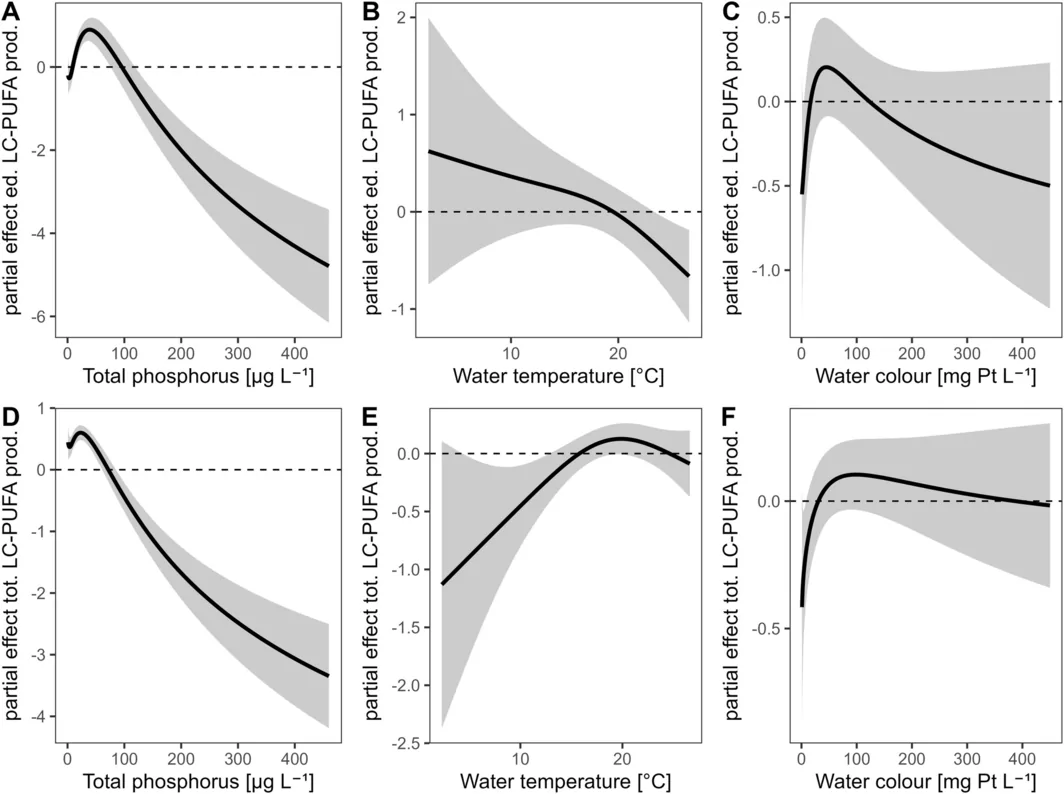
Effects of environmental change on phytoplankton LC‐PUFA biosynthesis across Finnish lakes. Partial effects from generalized additive mixed models showing the relationships between (A and D) total phosphorus, (B and E) temperature and (C and F) water colour with LC‐PUFA biosynthesis in the zooplankton‐edible phytoplankton fraction (< 35 μm; A–C) and total phytoplankton community (D–F). Lines show fitted smooth effects and shaded areas indicate 95% confidence intervals. Models included lake type as a categorical predictor and lake identity as a random effect to account for repeated summer observations. Biosynthesis units: Ng g C^−1^ h^−1^.

**FIGURE 5:**
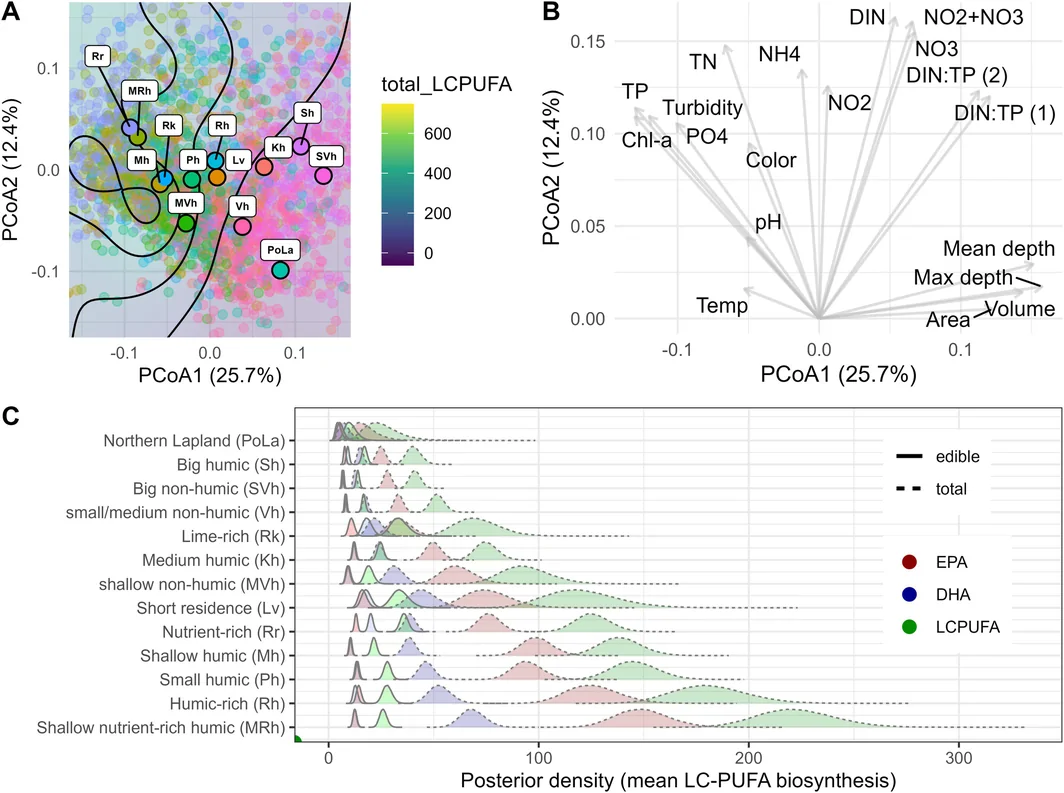
LC‐PUFA biosynthesis rates in different lake types. (A) A PCoA was used to evaluate the total LC‐PUFA primary biosynthesis [ng g C^−1^ h^−1^] in different lake types, with high productive lakes in the top left corner. (B) The corresponding eigenvectors of the PCoA explaining the lake dissimilarities. (C) Posterior distributions of the lake type effect on edible and total LC‐PUFA biosynthesis rates. For mode and 95% HDI see Table S3.

Geographical patterns in algal‐group dominance were observed in both total phytoplankton biomass and the most edible (< 35 μm) algal size fraction (Figure 4; Tables S5 and S6). In general, green algae and euglenoids were more dominant in southern and western lakes, whereas dinoflagellates, cryptomonads, and chrysophytes dominated in northern and eastern lakes. Large diatoms were also more dominant in northern lakes. Euglenoids were dominant in highly eutrophic, warmer, shallow, and darker lakes, while cryptomonads, diatoms, and chrysophytes dominated in deeper lakes. Finally, chrysophytes dominated in oligotrophic, colder, and clear water lakes (Table S5; Figure S2). Within the edible fraction (< 35 μm), raphidophytes were either absent or occurred at negligible levels, and no dominance was detected. LC‐PUFA biosynthesis rates significantly differed in the distinct lake types (Figure 5; Table S2), with Northern Lapland lakes having the lowest LC‐PUFA biosynthesis and the highest values found in naturally nutrient‐rich, lime‐rich, and short‐residence lakes. It is also notable that EPA biosynthesis exceeded DHA biosynthesis in all lake types (Figure 5C).

The absence of raphidophytes also significantly reduced the biosynthesis of LC‐PUFA in the most edible size class for zooplankton compared to the total phytoplankton. In the total phytoplankton fraction, out of the 643 lakes of the database, about 2/3 of lakes' LC‐PUFA biosynthesis rates were 100 ng g C^−1^ h^−1^ and above. In the < 35 μm size fraction, however, only 166 lakes (95% HDI: 84–248) had edible‐fraction LC‐PUFA biosynthesis rates above the isotope‐based threshold associated with strongly reduced consumer reliance on endogenous bioconversion. In approximately half of the lakes, predicted edible LC‐PUFA availability remained below the 50% isotope‐response threshold, indicating substantial potential reliance on endogenous bioconversion.

### 3.4 Environmental Drivers and Long‐Term Trends

Environmental gradients explained a substantial fraction of variation in phytoplankton LC‐PUFA biosynthesis, particularly in the zooplankton‐edible size fraction. The PCoA indicated that variation in edible LC‐PUFA production was embedded in a broader environmental gradient, with the strongest fitted vectors associated with lake depth, total phosphorus, chlorophyll‐a, dissolved inorganic nitrogen, lake area, and water color. Temperature was also significantly associated with the ordination, although its explanatory strength was lower than that of trophic‐state and morphometric variables.

In the GAMs, environmental predictors explained 35.8% of deviance in LC‐PUFA production of the edible phytoplankton fraction and 21.5% in total phytoplankton production. For the edible fraction, LC‐PUFA production was significantly related to total phosphorus, temperature, and water color (Figure 4). The partial effect of TP was unimodal, with the highest fitted values at approximately 39 μg TP L^−1^ and became negative above approximately 120 μg TP L^−1^, and predicted LC‐PUFA production declined strongly at high TP concentrations. Temperature had a weaker but significant effect, with reduced edible LC‐PUFA production at the warmest end of the observed gradient. Water color also affected edible LC‐PUFA production, although the response was less pronounced than for TP. Total nitrogen did not explain additional variation after accounting for the other environmental predictors.

Edible LC‐PUFA production showed a significant interaction between TP and temperature (Figure S1), indicating that temperature effects depended on lake trophic state. This suggests that warming does not have a uniform effect on nutritionally available LC‐PUFA production, but that its consequences are mediated by phosphorus availability. In contrast, total phytoplankton LC‐PUFA production was significantly related to TP, temperature, and water color, but the TP × temperature interaction was not supported. Total LC‐PUFA production showed a TP optimum at approximately 23 μg TP L^−1^ and declined at higher nutrient concentrations.

No consistent temporal trend was detected across the full lake dataset. When looking at lakes individually, however, the picture changed. To illustrate lake‐specific temporal trajectories, we selected three lakes with long and comparatively complete phytoplankton time series: Oulujärvi (64° N, area 928 km^2^), Vesijärvi (61° N, area 107 km^2^) and Tuusulanjärvi (60° N, area 6 km^2^), each with data since the 1980s. Lake Oulujärvi is a cold, northern, oligotrophic lake receiving nutrients mainly from its catchment and experienced only a negligible decrease in TP values over the decades. Vesijärvi was eutrophic in the 1980s due to sewage inputs; however, TP values have declined from ~50 to ~20 μg/L. Tuusulanjärvi was a hypereutrophic lake (TP > 150 μg/L) also due to sewage, but has stabilized at ~75 μg/L. Simultaneously, water temperature increased equally in all lakes during this period (~ + 0.07°C y^−1^). Based on the algal data available, we estimated that LC‐PUFA biosynthesis increased in Oulujärvi (+0.7 ng g C^−1^ h^−1^ y^−1^) and Tuusulanjärvi (+1.1 ng g C^−1^ h^−1^ y^−1^), while it decreased in Vesijärvi (−3.0 ng g^−1^ C h^−1^ y^−1^, Figure S3).

## 4 Discussion

### 4.1 Major Trends and Scope of Inference

This lake study demonstrates that eutrophication, warming, and browning jointly reshape phytoplankton community composition and biomass, thereby affecting the production and trophic transfer of LC‐PUFA of pelagic food‐web resources in boreal lakes. The LC‐PUFA biosynthesis varied strongly among phytoplankton groups, yet the most edible size fraction (< 35 μm) for zooplankton contained substantially less LC‐PUFA than the total phytoplankton pool. In many lakes, predicted LC‐PUFA production in the edible size fraction remained below isotope‐based thresholds associated with reduced consumer reliance on endogenous bioconversion, indicating that zooplankton and planktivorous fish increasingly depend on bioconversion pathways when dietary LC‐PUFA availability is low. LC‐PUFA production showed a unimodal relationship with TP concentrations, peaking at intermediate levels (~39 μg P L^−1^; minor eutrophication) and declining under high eutrophic conditions. This decline coincided with increasing dominance and biomass of cyanobacteria and green algae, taxa generally containing low LC‐PUFA contents. Temperature effects were not uniform but depended on trophic state, with the strongest TP‐dependent responses occurring in the most edible size fraction that directly supports secondary production. Browning further influenced dietary LC‐PUFA availability by modifying light climate, nutrient dynamics, and community composition, often favoring taxa with low nutritional quality. Together, these results reveal that eutrophication, warming, and lake browning as multiple global change stressors can constrain the dietary supply of essential biomolecules at the base of pelagic food webs, with consequences for consumer nutrition and aquatic food‐web functioning.

### 4.2 Phytoplankton Community Composition as the Basis of LC‐PUFA Production

These field measurements confirm that LC‐PUFA biosynthesis is strongly taxon‐dependent, consistent with laboratory studies showing large differences among algal groups in EPA and DHA production (Taipale et al. 2013; Taipale et al. 2020). High‐quality producers, including raphidophytes, dinoflagellates, cryptomonads, diatoms, and chrysophytes, were the main contributors to LC‐PUFA production, but their contribution differed between the total phytoplankton community and the most edible size fraction for zooplankton. Chrysophytes dominated the edible fraction in oligotrophic lakes, whereas dinoflagellates and cryptomonads were more important in meso‐ and eutrophic lakes. In contrast, green algae and euglenoids were negatively associated with community‐level LC‐PUFA biosynthesis, confirming these taxa have reduced EPA and DHA availability (Senar et al. 2019). These findings support previous evidence identifying dinoflagellates and cryptomonads as key vectors of LC‐PUFA transfer to higher trophic levels (Strandberg et al. 2015), and with experimental work showing that algal class identity explains far more variation in fatty‐acid profiles than environmental conditions such as nutrients or temperature (Calderini, Pääkkönen, et al. 2023; Galloway and Winder 2015). Our biosynthesis estimates therefore reflect how physicochemical conditions shape community composition and biomass, rather than species‐specific PUFA patterns alone.

Green algae were negatively associated with community‐level LC‐PUFA biosynthesis. Their limited ability to synthesize EPA and DHA likely reflects constraints in their desaturase/elongase pathways, which restrict production of C16 and C18 PUFA (Maltsev and Maltseva 2021; Taipale et al. 2013). Cyanobacteria similarly contributed little to LC‐PUFA production, although they may still support zooplankton indirectly through microbial pathways (Abonyi et al. 2023; de Kluijver et al. 2012). Euglenoids, despite their potential to synthesize LC‐PUFA under phototrophic conditions (Wang et al. 2018), were also negatively associated with LC‐PUFA production, potentially reflecting increased heterotrophic or mixotrophic metabolism in low‐light, DOM‐rich environments, which can reduce their LC‐PUFA content (Karnkowska et al. 2023; Wang et al. 2018). Similar responses have been reported for other mixotrophic flagellates (Shishlyannikov et al. 2014). Overall, our results indicate that community shifts toward green algae, euglenoids, or heterotrophic conditions reduce the basal production of EPA and DHA, whereas lakes dominated by cryptomonads, dinoflagellates, diatoms, or chrysophytes provide the highest quality of basal LC‐PUFA supply to pelagic consumers.

### 4.3 Interactive Effects of Eutrophication, Warming, and Browning of LC‐PUFA Supply

Our findings support the view that lake food quality is shaped by interacting global‐change pressures rather than by single environmental drivers. The strong influence of TP is consistent with previous studies showing that eutrophication increases total phytoplankton biomass while simultaneously reducing its nutritional quality (Calderini, Pääkkönen, et al. 2023; Taipale et al. 2016). In our study, LC‐PUFA production showed a unimodal relationship with TP: moderate nutrient availability supported productive LC‐PUFA‐synthesizing communities, whereas at high TP levels, increasing dominance of cyanobacteria, green algae, and other LC‐PUFA‐poor taxa reduced the overall contribution of EPA and DHA. Thus, eutrophication may increase primary production while simultaneously reducing the biochemical quality of the phytoplankton resource available to consumers at higher trophic levels.

The TP × temperature interaction in the most edible size fraction for zooplankton adds an important climate‐change dimension. Previous studies predicted that warming reduces phytoplankton n‐3 LC‐PUFA production, particularly EPA and DHA (Hixson and Arts 2016), although freshwater evidence from freshwater ecosystems suggests that warming effects are context‐dependent (Calderini, Kahilainen, et al. 2023). Our results support the latter view. Temperature effects were strongest on zooplankton‐accessible LC‐PUFA production and depended on TP rather than acting independently. Consequently, warming may have contrasting effects across oligotrophic and eutrophic lakes, with the strongest implications for trophic transfer because zooplankton selectively graze on the edible (< 35 μm) particle size fraction rather than on the entire phytoplankton community. Changes in the nutritional quality of this fraction are therefore likely to propagate efficiently through pelagic consumers.

Lake browning was an additional driver of LC‐PUFA dynamics, consistent with browning as a distinct axis of environmental change. Previous studies have shown that browning can reduce the availability of essential fatty acids and lower food‐web nutritional quality (Senar et al. 2019), although positive effects on phytoplankton biomass or LC‐PUFA concentrations have been reported under specific conditions (Strandberg et al. 2023). These observed contrasting responses likely reflect shifts in energy pathways from autotrophic to increasingly heterotrophic or mixotrophic production. By altering light climate, nutrient availability, microbial coupling, and phytoplankton community structure simultaneously, browning can change both the quantity and biochemical composition of basal resources. The significant effect of water color observed after accounting for TP and temperature gradients indicates that browning represents an independent axis of environmental change influencing the quantity and dietary quality of basal food webs. Because eutrophication, warming, and lake browning are expected to intensify in many northern lakes in the future, their combined effects may reduce trophic transfer efficiency, increase consumer reliance on endogenous LC‐PUFA synthesis, and ultimately alter ecosystem productivity and even resilience.

### 4.4 Consumer Bioconversion Responses and Limits of Trophic Attribution

Negative effects of eutrophication on LC‐PUFA content of herbivorous cladocerans and calanoids have been previously demonstrated in our 12 experimental lakes (Calderini, Pääkkönen, et al. 2023). Here, we focused on potential compensation mechanisms in herbivorous cladocerans. The isotope models indicated compensation by consumers when dietary EPA or DHA was low. In herbivorous cladocerans, δ^2^H‐ALA values closely tracked those of seston, indicating strong dietary coupling for this precursor of other n‐3 PUFA. In contrast, δ^2^H‐EPA values became progressively ^2^H—depleted as algal EPA production declined, pointing to increased endogenous conversion from EPA precursors. Such trophic upgrading by herbivores is crucial, as it buffers consumers at higher trophic levels against short‐term fluctuations in algal food quality (Senar et al. 2019). However, the extent of this buffering is likely taxon‐specific, as feeding strategies and the presence and regulation of desaturase genes differ markedly among aquatic consumers (Monroig et al. 2022), Our analyses focused mostly on herbivorous cladocerans, and responses of other zooplankton groups, for example, herbivorous calanoids, may differ substantially.

Increasing TP across these 12 studies revealed only weak effects of dietary LC‐PUFA availability on the LC‐PUFA content of planktivorous perch (Calderini, Pääkkönen, et al. 2023). Compound‐specific isotope values indicated an increased reliance on endogenous DHA biosynthesis under conditions of low dietary DHA supply. However, because zooplankton DHA contents were very low, reflecting the high contribution of daphnids, it was not possible to determine whether endogenous DHA biosynthesis occurred primarily in zooplankton, perch, or both. Consequently, DHA isotope patterns of DHA in fish integrate the nutritional dynamics of the pelagic food chain, including trophic transfer of preformed DHA and endogenous synthesis at various trophic consumer levels. This interpretation is supported by previous studies showing substantial contributions of daphnids to juvenile perch diet (Bretzel et al. 2021; Guma'a 1978) and by evidence that perch contain substantially higher LC‐PUFA contents than expected from dietary levels alone, indicating substantial endogenous bioconversion capacity (Scharnweber et al. 2021; Taipale et al. 2022). We thus interpret perch isotope data as evidence of increased consumer‐level bioconversion demand under low basal DHA production rather than as proof of perch‐specific DHA biosynthesis.

Expression of *fads2*, which encodes the Δ6 desaturase catalyzing the rate‐limiting step of LC‐PUFA biosynthesis (Geay et al. 2016), provided complementary evidence of activations of endogenous biosynthetic LC‐PUFA pathways (Ishikawa et al. 2019; Taipale et al. 2025). However, our results indicate that the *fads2* regulation is influenced by both nutritional and thermal conditions. In the covariate‐controlled model, *fads2* expression was more strongly associated with lake temperature than with LC‐PUFA from the most edible phytoplankton size fraction. This finding is consistent with previous evidence showing that fish may upregulate LC‐PUFA biosynthetic pathways in response to reduced dietary supply and to maintain membrane fluidity under changing thermal conditions (Bouaziz et al. 2017). Together, these consumer responses suggest that endogenous LC‐PUFA biosynthesis by zooplankton and planktivorous perch can partly compensate for low dietary EPA or DHA availability in pelagic food webs of boreal and subarctic lakes.

### 4.5 Lake‐Level Implications

Our survey of 643 lakes including 13 lake types shows that phytoplankton community composition and LC‐PUFA biosynthesis vary systematically across lake types and regions, with important consequences for the nutritional quality of pelagic food webs. Lakes in southern and western Finland were more frequently dominated by green algae and euglenoids, whereas northern and eastern lakes contained more cryptomonads, dinoflagellates, chrysophytes, and large diatoms, taxa known for their high EPA and DHA biosynthesis potential. These regional differences suggest that the ecological consequences of environmental change will vary among lake types. In oligotrophic systems, moderate nutrient enrichment and longer growing seasons may increase LC‐PUFA production; in contrast, further eutrophication, warming, or browning may shift communities toward taxa with lower LC‐PUFA. This agrees with previous observations of lower EPA and DHA contents in perch of eutrophic and brown lakes relative to oligotrophic clear‐water lakes (Taipale et al. 2016), although regional subarctic climatic gradients may modify these patterns (Keva et al. 2021).

Across the lake dataset, the modeled LC‐PUFA biosynthesis within the phytoplankton size fraction (< 35 μm) most edible by zooplankton was substantially lower than in the total phytoplankton size pool. Consequently, assessments based solely on total phytoplankton LC‐PUFA production may overestimate the dietary LC‐PUFA availability to herbivorous zooplankton and planktivorous fishes. Only 166 of the 643 lakes (~25%) exceeded the estimated LC‐PUFA production threshold associated with minimal consumer reliance on endogenous bioconversion. This infers that dietary LC‐PUFA availability is seasonally limited in many boreal and subarctic lakes, particularly where food web communities are dominated by taxa with low LC‐PUFA biosynthesis capacity. Nevertheless, these thresholds should be interpreted as indicators of pelagic LC‐PUFA availability rather than direct predictors of fish growth, survival, or fisheries production.

Our modeling framework focuses on pelagic phytoplankton‐zooplankton pathways and does not account for alternative LC‐PUFA inputs from benthic or littoral food webs. Littoral and benthic periphyton can contribute substantially to whole‐lake production, especially in small clear‐water and humic lakes (Vesterinen et al. 2017). However, benthic invertebrates generally contain lower LC‐PUFA contents than in zooplankton, which remain the principal dietary DHA source for fish consumers (Vesterinen et al. 2021). Quantifying the contribution of littoral and benthic pathways to ecosystem‐wide LC‐PUFA will require in situ measurements of LC‐PUFA production across habitat compartments.

Hixson and Arts (2016) have predicted substantial global declines in algal LC‐PUFA production under global warming scenarios, including reductions of ~8% of EPA and ~28% for DHA following a 2.5°C increase in water temperature. Our results suggest that temperature effects on LC‐PUFA production in boreal and subarctic lakes are strongly context dependent and mediated by trophic condition and community composition. While warmer lakes may currently support higher phytoplankton biomass and faster turnover rates, continued warming is expected to favor bloom‐forming cyanobacteria and other LC‐PUFA‐poor taxa, particularly in nutrient‐rich lakes. Thus, the effects of climate on pelagic LC‐PUFA production cannot be considered in isolation. Future changes in biochemical food‐web quality will likely depend on the joint effects of warming, nutrient loading, browning, lake morphometry, and shifts in phytoplankton community composition.

## 5 Conclusion

This study established a strong link between phytoplankton community composition and LC‐PUFA biosynthesis rates across boreal and subarctic lakes. The combination of detailed field measurements from 12 lakes with long‐term monitoring data from 643 lakes spanning broad temperature and total phosphorus gradients, we conclude that eutrophication, warming, and browning jointly structure phytoplankton communities and thereby alter the nutritional quality of pelagic food webs. Oligotrophic clear‐water lakes were generally dominated by LC‐PUFA‐rich taxa, whereas eutrophic and humic lakes contained PUFA‐poor communities. Across this study region, only ~25% of lakes exceeded isotope‐based production thresholds associated with minimal consumer reliance on endogenous LC‐PUFA bioconversion during summer. These findings indicate that dietary LC‐PUFA availability is constrained in many northern lakes, increasing the importance of endogenous LC‐PUFA biosynthesis by zooplankton and fishes. Finally, such trophic plasticity may partly buffer consumers against predicted declining food quality, but continued environmental global change is likely to intensify nutritional bottlenecks at the base of lake food webs, with consequences for lake productivity, fisheries, and food web resilience.

## Author Contributions

**Jussi Vesterinen:** methodology, writing – review and editing, resources. **Kristiina Vuorio:** methodology, writing – review and editing. **Matthias Pilecky:** conceptualization, writing – original draft, methodology, writing – review and editing, software, data curation. **Kimmo K. Kahilainen:** methodology, writing – review and editing. **Sami J. Taipale:** conceptualization, funding acquisition, writing – original draft, methodology, project administration, resources, supervision. **Cyril Rigaud:** methodology, writing – review and editing. **Timo J. Ruokonen:** methodology, writing – review and editing, resources. **Marco Calderini:** methodology, writing – review and editing. **Martin J. Kainz:** methodology, writing – review and editing, resources.

## Funding

The present study was funded by the Research Council of Finland (project 371187 granted to Sami J. Taipale and project 367980 granted to Kristiina Vuorio) and by the Austrian Science Fund (project PAT6359323 – “HydroFAT” granted to Martin J. Kainz).

## Conflicts of Interest

The authors declare no conflicts of interest.

## Supporting information


**Data S1:** gcb71070‐sup‐0001‐DataS1.pdf.

## Data Availability

Phytoplankton data are available at open‐access interfaces for environmental data at the Finnish Environmental Centre (www.syke.fi/en/environmental‐data). The hydrogen‐isotope data of fatty acids from seston, zooplankton, and planktivorous fish are available in the DOOR repository (https://doi.org/10.48341/47nm‐qr72), and all other data can be found in the Supporting Information.
