# Supplementary material for: Global Change Reshapes Biosynthesis of LC‐PUFA in Northern Lake Food Webs
Source: Glob Chang Biol. 2026 Aug 24;32(8):e71070. doi: 10.1111/gcb.71070 (PMC13501198; doi:10.1111/gcb.71070)
Supplement: Supplementary file 1 [file GCB-32-e71070-s001.pdf]

Global change reshapes biosynthesis of LC-PUFA in northern lake food webs

Matthias Pilecky[1,2*], Kristiina Vuorio[3], Cyril Rigaud[4], Marco Calderini[4], Kimmo K. Kahilainen[5], Timo J. Ruokonen[6], Martin J. Kainz[1,2], Jussi Vesterinen[7], Sami J. Taipale[4*]

Supplement Material

1) WasserCluster Lunz – Biologische Station, Dr. Carl-Kupelwieser Promenade 5, 3293 Lunz am See, Austria

2) Universität für Weiterbildung Krems, Research Lab for Aquatic Ecosystem Research and -Health, Dr. Karl-Dorrek-Straße 30, 3500 Krems, Austria

3) Finnish Environment Institute, Latokartanonkaari 9, FI-00790 Helsinki, Finland

4) University of Jyväskylä, Department of Biological and Environmental Science, Survontie 9C, 40014, Jyväskylä, Finland

5) Lammi Biological Station, University of Helsinki, Pääjärventie 320, FI-16900 Lammi, Finland

6) Natural Resources Institute Finland (Luke), Latokartanonkaari 9, FI-00790 Helsinki, Finland

*equal contribution / corresponding authors

E-mail:

MP: matthias.pilecky@donau-uni.ac.at

ST: sami.taipale@jyu.fi

Running Headline: Linking phytoplankton with upper trophic levels

Table S1. Median, minimum and maximum of biomass of phytoplankton biomass (μg C L^{-1}) in the experimental lake data (n = 12) and in Hertta data (n = 643 lakes, and n = 9225 samples).

		Cyanobacteria	Green algae	Raphidophytes	Golden algae	Cryptophyceae	Diatoms	Dinoflagellates
**Experimental lakes**	Min	0	3	0	0	5	2	0
	Max	4366	400	93	251	734	1066	158
**HERTTA database**	Median	9.6	9	0.09	16.9	17.9	17.8	5.1
	Min	0	0	0	0	0	0	0
	Max	13177	2683	2789	1774	1214	4012	4697
	3rd Quarter	41.6	22.1	7	32	39	48	12

Table S2. Gut-content and fads2 and elolv5 gene expression of studied perch.

Sample	Species	Lake	Type	Class	fads2	elovl5	gut content	Decomposition stage	Stomach fullness	Chironomid pupae	Cladocerans	Copepoda	Insect larvae	Fish remains	Surface insects	Cyprinid fish	Daphnia	Mysis	Cyclopoida	Leptodora	Calanoida	Ephemeroptera nymph	Bosmina	Chaoborus larvae	Diaphanosoma	Chironomid larvae	Zooplankton	Pikeperch	Trichoptera larvae	Eurycercus	Odonata nymph	Bleak	Asellus
ENAP02	Perch	Enäjärvi	Eutrophic	Medium	0.8	0.7	no																										
21HPE01	Perch	Hulausjärvi	Eutrophic	Medium	0.37	0.3	yes	2	2	2																							
21HPE02	Perch	Hulausjärvi	Eutrophic	Small	1.19	0.3	yes	2	1		1																						
21HPE03	Perch	Hulausjärvi	Eutrophic	Small	0.88	1.5	yes	2	2		2																						
21HPE04	Perch	Hulausjärvi	Eutrophic	Small	0.96	1.4	yes	2	2		1	1																					
21HPE05	Perch	Hulausjärvi	Eutrophic	Medium	0.18	0.1	yes		0																								
21HPE06	Perch	Hulausjärvi	Eutrophic	Medium	0.36	0	yes		0																								
21HPE07	Perch	Hulausjärvi	Eutrophic	Medium	0.36	0.4	yes		0																								
21HPE08	Perch	Hulausjärvi	Eutrophic	Small	1.12	1.2	yes	3	3		1		2																				
21HPE09	Perch	Hulausjärvi	Eutrophic	Small	0.64	0.4	yes		0																								
21HPE10	Perch	Hulausjärvi	Eutrophic	Small	1.38	2.9	yes		0																								
21HPE11	Perch	Hulausjärvi	Eutrophic	Small	0.91	0.7	yes	3	1		1																						
ISOPE01	Perch	Isojärvi	Oligotrophic	Medium	1.66	2.1	nd																										
ISOPE05	Perch	Isojärvi	Oligotrophic	Medium	2.22	3.6	nd																										
ISOPE06	Perch	Isojärvi	Oligotrophic	Medium	1.48	2.3	nd																										
ISOPE07	Perch	Isojärvi	Oligotrophic	Medium	1.67	1.3	nd																										
ISOPE08	Perch	Isojärvi	Oligotrophic	Small	0.95	0.2	nd																										
ISOPE09	Perch	Isojärvi	Oligotrophic	Medium	1.63	2.1	nd																										
ISOPE10	Perch	Isojärvi	Oligotrophic	Medium	1.1	1	nd																										
ISOPE11	Perch	Isojärvi	Oligotrophic	Small	0.86	0.5	nd																										
ISOPE12	Perch	Isojärvi	Oligotrophic	Small	1.57	1.8	nd																										
ISOPE13	Perch	Isojärvi	Oligotrophic	Small	1.4	1.7	nd																										
ISOPE14	Perch	Isojärvi	Oligotrophic	Medium	1.54	2.1	nd																										
21KJPE01	Perch	Korpijärvi	Oligotrophic	Medium	1.37	0.4	yes		0																								
21KJPE03	Perch	Korpijärvi	Oligotrophic	Medium	1.36	0.7	yes		0																								
21KJPE04	Perch	Korpijärvi	Oligotrophic	Medium	1.04	1.4	yes	3	1					1																			
21KJPE05	Perch	Korpijärvi	Oligotrophic	Medium	1.08	0.5	yes	2	4						2	2																	
21KJPE06	Perch	Korpijärvi	Oligotrophic	Medium	1.64	2	yes	2	3								2.5	0.5															
21KJPE07	Perch	Korpijärvi	Oligotrophic	Small	1.34	1.1	yes	2	4								3.5		0.5														
21KJPE08	Perch	Korpijärvi	Oligotrophic	Small	1.27	0.9	yes	2	5						5																		
21KJPE09	Perch	Korpijärvi	Oligotrophic	Small	2.2	1.9	yes		0																								
21KJPE10	Perch	Korpijärvi	Oligotrophic	Small	1.44	2	yes	2	7								3				4												
21KJPE11	Perch	Korpijärvi	Oligotrophic	Small	1.34	0.9	yes		0																								
21KJPE12	Perch	Korpijärvi	Oligotrophic	Small	1.68	2.1	yes	2	8						2		1				5												
21KKPE01	Perch	Kukkia	Oligotrophic	Medium	1.19	0.2	yes	2	2														2										
21KKPE02	Perch	Kukkia	Oligotrophic	Medium	1.05	3.1	yes	3	1		1																						
21KKPE03	Perch	Kukkia	Oligotrophic	Medium	1.05	0.7	yes	2	1														1										
21KKPE04	Perch	Kukkia	Oligotrophic	Medium	0.49	0.1	yes		0																								
21KKPE05	Perch	Kukkia	Oligotrophic	Medium	1.52	2	yes		0																								

21KKPE06	Perch	Kukkia	Oligotrophic	Medium	0.9	0.3	yes	2	3								3																
21KKPE07	Perch	Kukkia	Oligotrophic	Medium	1.22	1.9	yes	2	6								4				2												
21KKPE08	Perch	Kukkia	Oligotrophic	Small	1.22	1.8	yes	3	2		2																						
21KKPE09	Perch	Kukkia	Oligotrophic	Small	1.56	2.1	yes	2	6								5				1												
21KKPE10	Perch	Kukkia	Oligotrophic	Small	1.08	1.7	yes	2	3														3										
21KKPE11	Perch	Kukkia	Oligotrophic	Small	0.65	0.4	yes		0																								
21KKPE12	Perch	Kukkia	Oligotrophic	Small	1.38	1.6	yes	2	5								3				1	1											
LOHP02	Perch	Lohjanjärvi	Mesotrophic	Medium	0.37	0.5	nd																										
LOHP03	Perch	Lohjanjärvi	Mesotrophic	Small	0.6	0.6	nd																										
LOHP04	Perch	Lohjanjärvi	Mesotrophic	Medium	0.75	0.6	nd																										
LOHP05	Perch	Lohjanjärvi	Mesotrophic	Small	0.59	0.7	nd																										
LOHP06	Perch	Lohjanjärvi	Mesotrophic	Small	0.8	2.6	nd																										
LOHP07	Perch	Lohjanjärvi	Mesotrophic	Small	0.99	2.4	nd																										
LOHP08	Perch	Lohjanjärvi	Mesotrophic	Small	1.02	1.9	nd																										
21MPE01	Perch	Majajärvi	Eutrophic	Small	0.01	0	yes	2	2														0.6	1	0.4								
21MPE02	Perch	Majajärvi	Eutrophic	Small	2.23	6.9	yes	3	1														1										
21MPE03	Perch	Majajärvi	Eutrophic	Small	1.73	3.3	yes		0																								
21MPE04	Perch	Majajärvi	Eutrophic	Small	4.58	4.6	yes	2	+																+								
21MPE05	Perch	Majajärvi	Eutrophic	Small	1.05	1.4	yes	3	6															6									
21MPE06	Perch	Majajärvi	Eutrophic	Small	1.37	1	yes	4	1															1									
21MPE07	Perch	Majajärvi	Eutrophic	Small	1.65	1.2	yes	3	8			2												6									
21MPE08	Perch	Majajärvi	Eutrophic	Small	1.14	0.8	yes	2	6															6									
21MPE09	Perch	Majajärvi	Eutrophic	Small	1.45	1.6	yes	2	9															9									
21MPE10	Perch	Majajärvi	Eutrophic	Small	2.21	3.9	yes	3	2			1												0.6		0.4							
21PPE702	Perch	Pääjärvi	Oligotrophic	Medium	0.76	0.7	yes	3	3					3																			
21PPE703	Perch	Pääjärvi	Oligotrophic	Small		NA	yes	1	4								2				2												
21PPE704	Perch	Pääjärvi	Oligotrophic	Small	0.97	1	yes		0																								
21PPE705	Perch	Pääjärvi	Oligotrophic	Small	1.26	1.8	yes	2	7								5		1		1												
21PPE706	Perch	Pääjärvi	Oligotrophic	Small	1.64	2.4	yes	1	8								6				2												
21PPE707	Perch	Pääjärvi	Oligotrophic	Small	1.22	1.1	yes	2	8			4					4																
21PPE708	Perch	Pääjärvi	Oligotrophic	Small	2	2.8	yes	2	7			2					5																
21PPE709	Perch	Pääjärvi	Oligotrophic	Small	2.05	2.9	yes	1	8								5				3												
21PPE710	Perch	Pääjärvi	Oligotrophic	Small	2.56	1.4	yes	2	5								4				1												
21PPE721	Perch	Pääjärvi	Oligotrophic	Small	0.94	0.6	yes	2	2						2																		
21PPE722	Perch	Pääjärvi	Oligotrophic	Small	1.41	1.3	yes	3	3			0.3		2.7																			
21PPE723	Perch	Pääjärvi	Oligotrophic	Medium	0.75	1.8	yes	2	1								0.5				0.5												
21TPE01	Perch	Tuusulanjärvi	Eutrophic	Small	0.29	0	yes		0																								
21TPE02	Perch	Tuusulanjärvi	Eutrophic	Medium	0.33	0	yes	2	7					7																			
21TPE03	Perch	Tuusulanjärvi	Eutrophic	Small	0.42	0.7	yes		+																		+						
21TPE04	Perch	Tuusulanjärvi	Eutrophic	Medium	0.62	0.6	yes	3	2					2																			
21TPE05	Perch	Tuusulanjärvi	Eutrophic	Small	0.45	0.5	yes	2	8																			8					
21TPE06	Perch	Tuusulanjärvi	Eutrophic	Medium	0.48	0.6	yes		0																								
21TPE07	Perch	Tuusulanjärvi	Eutrophic	Small	0.75	0.8	yes	3	2					2																			
21TPE08	Perch	Tuusulanjärvi	Eutrophic	Medium	0.24	0.3	yes		0																								
21TPE09	Perch	Tuusulanjärvi	Eutrophic	Small	1.04	1	yes	2	4											4													
21TPE10	Perch	Tuusulanjärvi	Eutrophic	Small	0.31	0.1	yes		0																								
21TPE11	Perch	Tuusulanjärvi	Eutrophic	Small	0.4	0.3	yes		0																								
21TPE12	Perch	Tuusulanjärvi	Eutrophic	Small	1.38	1.2	yes		0																								

21TPE13	Perch	Tuusulanjärvi	Eutrophic	Small	1.58	5.6	yes		+																		+						
21VKPE02	Perch	Valkea-Kotinen	Oligotrophic	Medium	1.15	1.2	yes	1	5																						5		
21VKPE03	Perch	Valkea-Kotinen	Oligotrophic	Medium	0.83	0.4	yes	2	2																						2		
21VKPE04	Perch	Valkea-Kotinen	Oligotrophic	Medium	1.4	1.4	yes	3	3				3																				
21VKPE05	Perch	Valkea-Kotinen	Oligotrophic	Medium	0.77	1	yes	2	2																						2		
21VKPE06	Perch	Valkea-Kotinen	Oligotrophic	Medium	1.49	4.3	yes	2	2																				1.5	0.5			
21VKPE07	Perch	Valkea-Kotinen	Oligotrophic	Medium	2.72	2.5	yes	2	4															4									
21VPE01	Perch	Vesijärvi	Mesotrophic	Medium	2.71	2.9	yes	2	2																								2
21VPE02	Perch	Vesijärvi	Mesotrophic	Medium	0.46	0.1	yes	3	3	1				2																			
21VPE03	Perch	Vesijärvi	Mesotrophic	Medium	0.29	0	yes		0																								
21VPE04	Perch	Vesijärvi	Mesotrophic	Medium	1.09	0.1	yes	2	5																							5	
21VPE05	Perch	Vesijärvi	Mesotrophic	Medium	0.83	1	yes		0																								
21VPE06	Perch	Vesijärvi	Mesotrophic	Medium	0.58	0.7	yes		0																								
21VPE12	Perch	Vesijärvi	Mesotrophic	Small	0.85	0.8	yes		0																								
21VPE13	Perch	Vesijärvi	Mesotrophic	Small	0.24	0	yes	2	3	3																							
21VPE14	Perch	Vesijärvi	Mesotrophic	Small	1.05	0.9	yes	3	1	1																							
21VPE15	Perch	Vesijärvi	Mesotrophic	Small	0.88	1.6	yes	2	2													1				1							
21VPE16	Perch	Vesijärvi	Mesotrophic	Small	0.88	2.2	yes	2	1													1											
21VPE17	Perch	Vesijärvi	Mesotrophic	Small	1.12	2.5	yes	2	3	1	2																						
21VPE25	Perch	Vesijärvi	Mesotrophic	Medium	1.59	2	yes	4	3					3																			
21VPE26	Perch	Vesijärvi	Mesotrophic	Medium	1.17	1.4	yes		0																								
21VPE27	Perch	Vesijärvi	Mesotrophic	Medium	0.83	1.1	yes	3	2											1.9			0.1										
21VPE28	Perch	Vesijärvi	Mesotrophic	Medium	1.29	0.6	yes		0																								
21VPE29	Perch	Vesijärvi	Mesotrophic	Medium	2.23	4.1	yes	4	1					1																			
21VPE30	Perch	Vesijärvi	Mesotrophic	Small		NA	yes	3	1		1																						
21VPE31	Perch	Vesijärvi	Mesotrophic	Small	0.79	1.5	yes	2	1						1																		
21VPE32	Perch	Vesijärvi	Mesotrophic	Small	0.73	1.2	yes		0																								
21VPE33	Perch	Vesijärvi	Mesotrophic	Small	0.94	1.6	yes		0																								
21VPE34	Perch	Vesijärvi	Mesotrophic	Small	1.1	1.4	yes		0																								
21VPE35	Perch	Vesijärvi	Mesotrophic	Small	1.12	1.5	yes	2	1														1										
21VPE36	Perch	Vesijärvi	Mesotrophic	Small	2.41	4	yes	1	+														+										
VIKPE06	Perch	Vikträsk	Eutrophic	Medium	0.78	2.1	nd																										
VIKPE07	Perch	Vikträsk	Eutrophic	Medium	0.67	1.5	nd																										
VIKPE08	Perch	Vikträsk	Eutrophic	Small	1.01	1.9	nd																										
VIKPE09	Perch	Vikträsk	Eutrophic	Small	1.24	2.2	nd																										
VIKPE10	Perch	Vikträsk	Eutrophic	Small	0.82	1.1	nd																										
VIKPE11	Perch	Vikträsk	Eutrophic	Small	1.28	2	nd																										
VIKPE12	Perch	Vikträsk	Eutrophic	Small	1.03	2.3	nd																										
VIKPE13	Perch	Vikträsk	Eutrophic	Small	0.79	1.4	nd																										

Table S3. Biosynthesis rates of LC-PUFA for each algae. Values are given as posterior medians [95 % HDI].

	EPA	DHA	LCPUFA
**Raphidophytes**	1874 [1720; 2027]	975 [821; 1128]	2849 [2542; 3155]
**Cyanobacteria**	-2 [-7; 0]	-14 [-19; -9]	-16 [-26; -9]
**Green algae**	-96 [-157; -19]	-23 [-86; 0]	-119 [-242; -19]
**Golden Algae**	567 [513; 622]	105 [50; 160]	673 [563; 782]
**Cryptomonads**	124 [90; 157]	440 [406; 473]	564 [496; 631]
**Diatomes**	931 [903; 955]	114 [95; 139]	1045 [998; 1094]
**Dinoflagellates**	346 [207; 483]	696 [561; 832]	1042 [768; 1315]
**Euglenoids**	-389 [-433; -346]	-264 [-307; -221]	-653 [-739; -567]

Table S4. Stable hydrogen isotope (δ^2H) values of seston, bulk zooplankton and perch. Values are given as mean ± sd.

	Seston			Zooplankton			Juvenile perch		
	ALA	EPA	DHA	ALA	EPA	DHA	ALA	EPA	DHA
**Enäjärvi**	-236.4 ± 17.0	-298.3 ± 10.1	NA	-220.6 ± 4.8	-331.0 ± 12.1	-188.7 ± 3.5	-326.50 ± NA	-272.08 ± NA	-293.8 ± NA
**Hulausjärvi**	-235.4 ± 7.7	-303.7 ± 3.8	-229.4 ± 11.3	-164.7 ± 15.8	-340.1 ± 14.7	-182.8 ± NA	-181.5 ± 9.5	-282.2 ± 1.9	-297.8 ± 2.4
**Isojärvi**	-189.5 ± 23.9	-296.0 ± 9.3	-258.2 ± 48.7	-169.8 ± 8.8	-321.3 ± 1.9	-252.54 ± NA	-240.1 ± 20.8	-288.2 ± 8.1	-297.7 ± 2.4
**Korpijärvi**	-158.6 ± 9.2	-310.9 ± 6.5	-248.7 ± 1.7	-150.2 ± 8.8	-323.6 ± 4.3	-224.8 ± 3.4	-248.9 ± 40.6	-282.1 ± 4.0	-293.5 ± 1.5
**Kukkia**	-201.6 ± 11.8	-319.6 ± 10.2	-262.1 ± 32.2	-196.6 ± 12.8	-333.3 ± 17.8	-202.4 ± NA	-211.9 ± 43.9	-290.7 ± 4.2	-310.7 ± 7.5
**Lohjanjärvi**	-228.9 ± 3.8	-328.1 ± 10.7	-293.9 ± 17.0	-206.2 ± 15.5	-343.8 ± 12.2	-217.4 ± 9.8	-272.7 ± 16.9	-290.0 ± 9.4	-314.0 ± 5.5
**Majajärvi**	-187.7 ± 14.7	-278.7 ± 3.6	NA	-191.0 ± 17.4	-317.1 ± 28.3	NA	-230.2 ± 37.0	-283.8 ± 5.9	-300.2 ± 2.6
**Pääjärvi**	-209.6 ± 24.7	-323.4 ± 22.8	-221.4 ± 2.8	-204.8 ± 19.6	-330.7 ± 10.2	-260.2 ± 6.0	-188.8 ± 10.8	-304.2 ± 2.8	-316.6 ± 5.8
**Tuusulanjärvi**	-238.9 ± 12.3	-342.7 ± 6.8	-268.3 ± 10.8	-188.5 ± 35.3	-346.5 ± 3.2	-211.5 ± NA	-168.4 ± 35.7	-296.7 ± 9.4	-308.5 ± 8.9
**Valkea-Kotinen**	-190.2 ± 43.8	-269.0 ± 39.0	-257.1 ± 32.1	-127.5 ± 11.5	-296.2 ± 11.7	-204.1 ± 13.5	-115.1 ± 13.0	-264.5 ± 5.0	-258.5 ± 5.4
**Vesijärvi**	-212.2 ± 17.0	-319.7 ± 5.0	-265.5 ± 1.7	-186.3 ± 15.6	-347.5 ± 14.3	-237.4 ± NA	-255.4 ± 12.1	-290.6 ± 3.5	-316.2 ± 4.4
**Viträsk**	-227.8 ± 36.5	-293.4 ± 40.2	-255.1 ± 52.4	-171.3 ± 34.5	-341.9 ± 7.6	-189.6 ± NA	-259.1 ± 8.3	-291.0 ± 2.8	-312.0 ± 3.8

Table S5. Posterior (Mode + 95 % HDI) of the mean lake type effects on PUFA biosynthesis rates [ng g C^{-1} h^{-1}].

	Obs.	n	DHA (edible)	DHA (total)	EPA (edible)	EPA (total)	LCPUFA (edible)	LCPUFA (total)
Big humic (Sh)	1165	33	9.09 [7.85; 10.5]	14.9 [12.5; 18]	7.64 [6.63; 8.86]	24.8 [20.6; 29.4]	17.1 [14.7; 19.5]	40 [33.8; 47.5]
Big non-humic (SVh)	2055	52	6.52 [5.8; 7.3]	12.6 [10.9; 14.3]	7.05 [6.34; 7.91]	28 [24.5; 31.8]	13.9 [12.5; 15.5]	41.2 [36; 46.2]
Humic-rich (Rh)	412	30	12.8 [10.8; 15]	52.1 [42.7; 63.6]	14.3 [12.2; 16.8]	125 [102; 151]	28.1 [23.6; 32.5]	179 [148; 217]
Lime-rich (Rk)	167	16	17.8 [14.1; 23.2]	21.7 [15.7; 29.8]	10.8 [8.42; 13.9]	33 [23.9; 45.2]	33.3 [26; 42]	68.8 [50.3; 92]
Medium humic (Kh)	858	57	12.3 [10.9; 13.8]	23.8 [20.5; 27.6]	11.9 [10.6; 13.5]	49.7 [42.8; 57.5]	24.7 [21.9; 27.6]	74.5 [64.8; 85.7]
Nutrient-rich (Rr)	1342	80	20.2 [18; 22.3]	38.5 [33.9; 44.2]	13.1 [11.7; 14.5]	76.2 [66.3; 86.7]	35.7 [32.3; 39.8]	124 [110; 142]
Polar and Lapland (PoLa)	27	4	4.26 [2.2; 7.82]	6.72 [3.09; 14]	5.22 [2.78; 9.25]	14.7 [7.42; 29.8]	9.81 [5.45; 16.7]	22.6 [11.4; 42.5]
Shallow humic (Mh)	621	85	10.3 [9.09; 11.6]	38.3 [33.1; 44.4]	10.5 [9.35; 11.9]	97.8 [85.1; 114]	21.3 [19.2; 24.1]	137 [119; 158]
Shallow nutrient-rich humic (MRh)	511	64	12.5 [10.9; 14.4]	68 [57.6; 80.3]	12.3 [10.8; 14.2]	149 [125; 174]	25.8 [22.6; 29.4]	219 [187; 259]
Short residence (Lv)	186	12	17.6 [13.8; 22.5]	43.5 [32.2; 58.6]	15.9 [12.5; 20.4]	74.2 [54.9; 98.2]	33.5 [26.6; 42.6]	118 [88.3; 155]
Small humic (Ph)	688	85	13.2 [11.8; 15]	45.9 [39.7; 53.8]	13.7 [12.2; 15.5]	93.2 [80.7; 110]	27.9 [24.9; 31.3]	144 [125; 167]
Shallow non-humic (MVh)	131	24	9.41 [7.52; 11.7]	30.9 [23.9; 40.4]	9.04 [7.23; 11.3]	60.2 [46.5; 77.7]	19.3 [15.4; 23.4]	91.8 [71.8; 118]
Small/medium non-humic (Vh)	1062	103	7.92 [7.08; 8.77]	17.4 [15.4; 19.9]	8.39 [7.56; 9.28]	33.1 [29.2; 37.8]	16.6 [15.1; 18.4]	51.3 [45.6; 58.3]

Obs. = Number of total Observations lake x sampling years; n = number of lakes in that category

Table S6. Optimal Emmeans ± SE of environmental variables propagating dominance of specific algal groups.

	TP [µg L^{-1}]		TN [µg L^{-1}]		Temperature [°C]		Color (mg Pt L^{-1})		Latitude (N°)		Longitude (E°)		Max depth [m]	
	**total**	**<35**	**total**	**<35**	**total**	**<35**	**total**	**<35**	**total**	**<35**	**total**	**<35**	**total**	**<35**
Raphidophytes	28.2 ± 2.2	NA	547 ± 27	NA	22.0 ± 0.2	NA	104 ± 5	NA	62.1 ± 0.2	NA	25.8 ± 0.2	NA	12.0 ± 1.8	NA
Cryptomonads	26.6 ± 3.1	29.3 ± 1.4	588 ± 39	590 ± 17	22.8 ± 0.3	22.6 ± 0.1	66 ± 7	72 ± 3	61.9 ± 0.3	62.4 ± 0.1	26.1 ± 0.3	26.2 ± 0.1	38.5 ± 2.4	23.7 ± 1.1
Cyanobacteria	51.6 ± 1.9	58.4 ± 18.4	848 ± 23	1044 ± 224	22.8 ± 0.2	23.2 ± 1.7	72 ± 4	66 ± 37	62.3 ± 0.2	61.9 ± 1.4	25.5 ± 0.2	24.1 ± 1.5	11.0 ± 1.5	3.8 ± 18.7
Diatoms	25.0 ± 3.0	32.8 ± 7.8	512 ± 37	611 ± 96	21.9 ± 0.3	21.4 ± 0.7	86 ± 6	129 ± 16	63.1 ± 0.2	62.2 ± 0.6	26.8 ± 0.3	25.2 ± 0.6	25.1 ± 2.2	18.4 ± 6.2
Dinoflagellates	34.4 ± 13.8	25.5 ± 6.0	517 ± 173	549 ± 73	23.7 ± 1.4	22.1 ± 0.5	63 ± 29	59 ±12	61.4 ± 1.1	63.0 ± 0.4	26.6 ± 1.2	25.7 ± 0.5	11.7 ± 6.7	13.5 ± 5.0
Euglenoids	79.7 ± 11.9	62.3 ± 5.7	729 ± 150	817 ±69	24.8 ± 1.2	22.9 ± 0.5	119 ± 25	95 ± 12	60.5 ± 1.0	61.4 ± 0.4	24.3 ± 1.1	24.6 ± 0.5	4.4 ± 6.7	6.1 ± 5.0
Golden algae	13.4 ± 2.0	17.5 ± 2.5	412 ± 25	419 ± 30	21.9 ± 0.2	21.5 ± 0.2	48 ± 4	69 ± 5	62.8 ± 0.2	63.0 ± 0.2	26.2 ± 0.2	26.1 ± 0.2	29.3 ± 1.6	19.3 ± 2.0
Green algae	17.7 ± 5.1	50.6 ± 3.2	486 ± 64	877 ± 39	23.3 ± 0.5	22.6 ± 0.3	58 ± 11	75 ±7	61.1 ± 0.4	61.5 ± 0.3	25.3 ± 0.5	24.9 ± 0.3	13.1 ± 4.2	8.4 ± 2.9

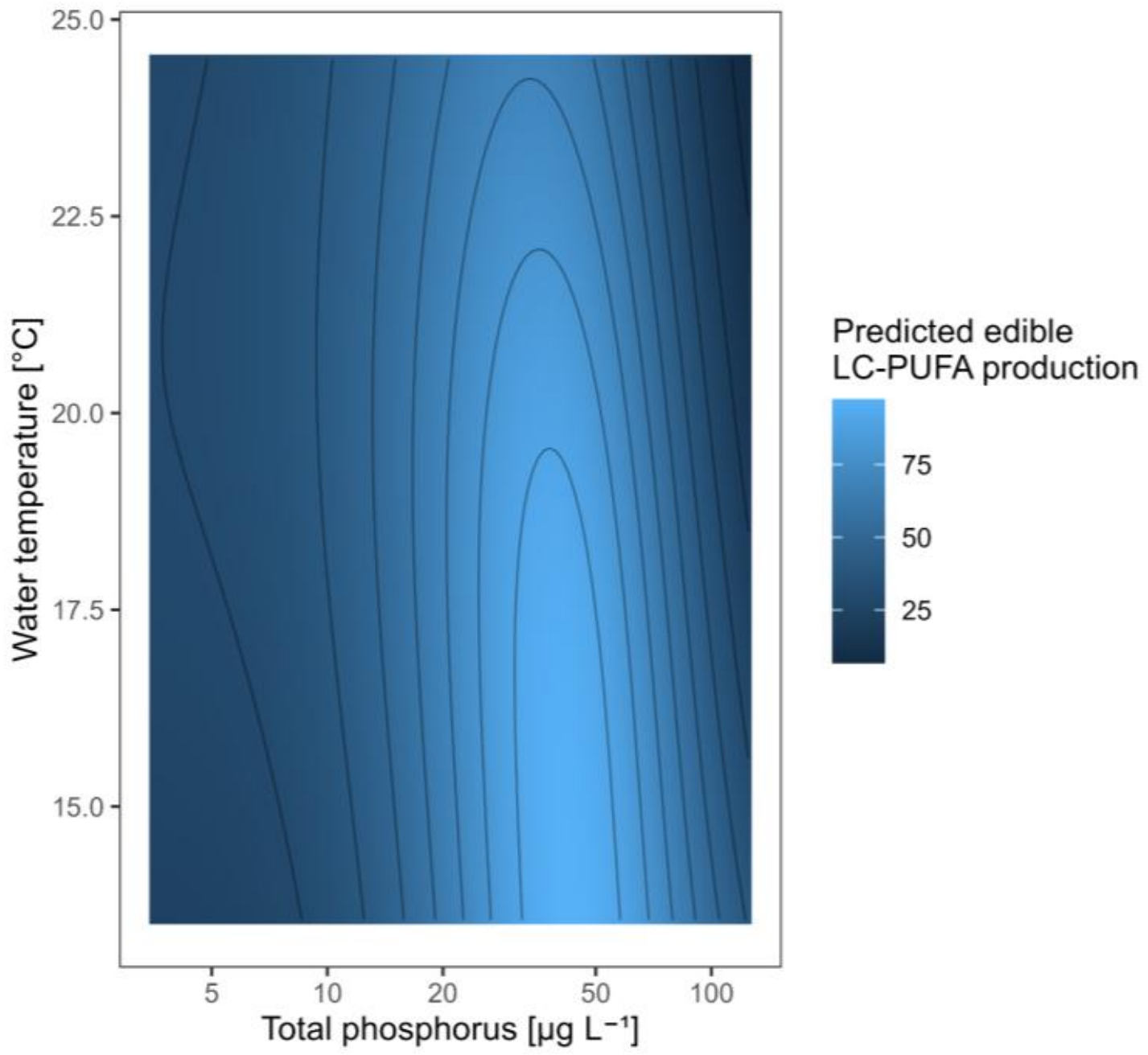

Figure S1. Interaction of temperature and total phosphorus effects on edible LC-PUFA biosynthesis in 643 boreal lakes.

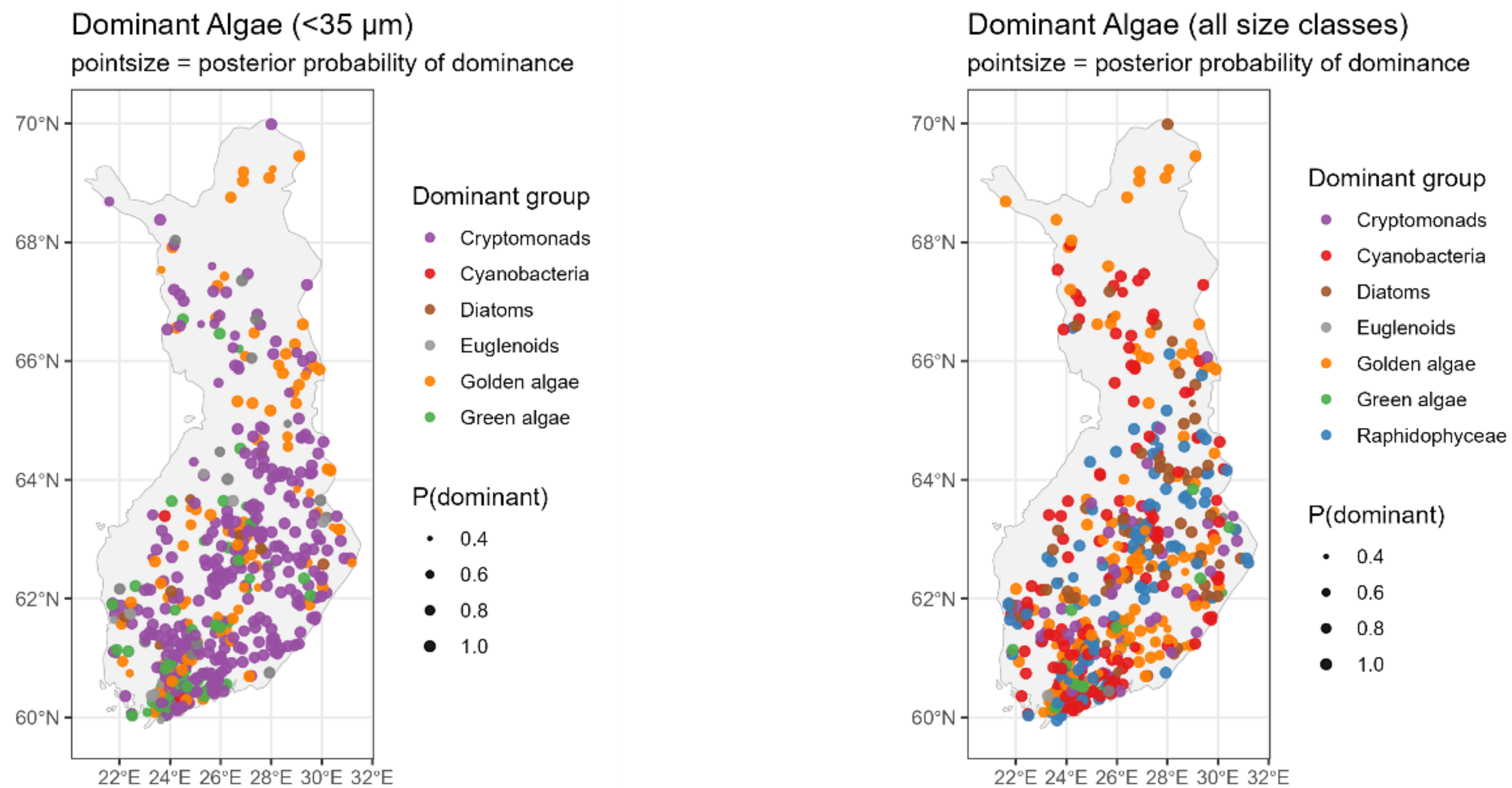

Figure S2. Dominance of algae groups for 643 boreal and subarctic lakes from publicly open data of the Finnish Environmental Centre.

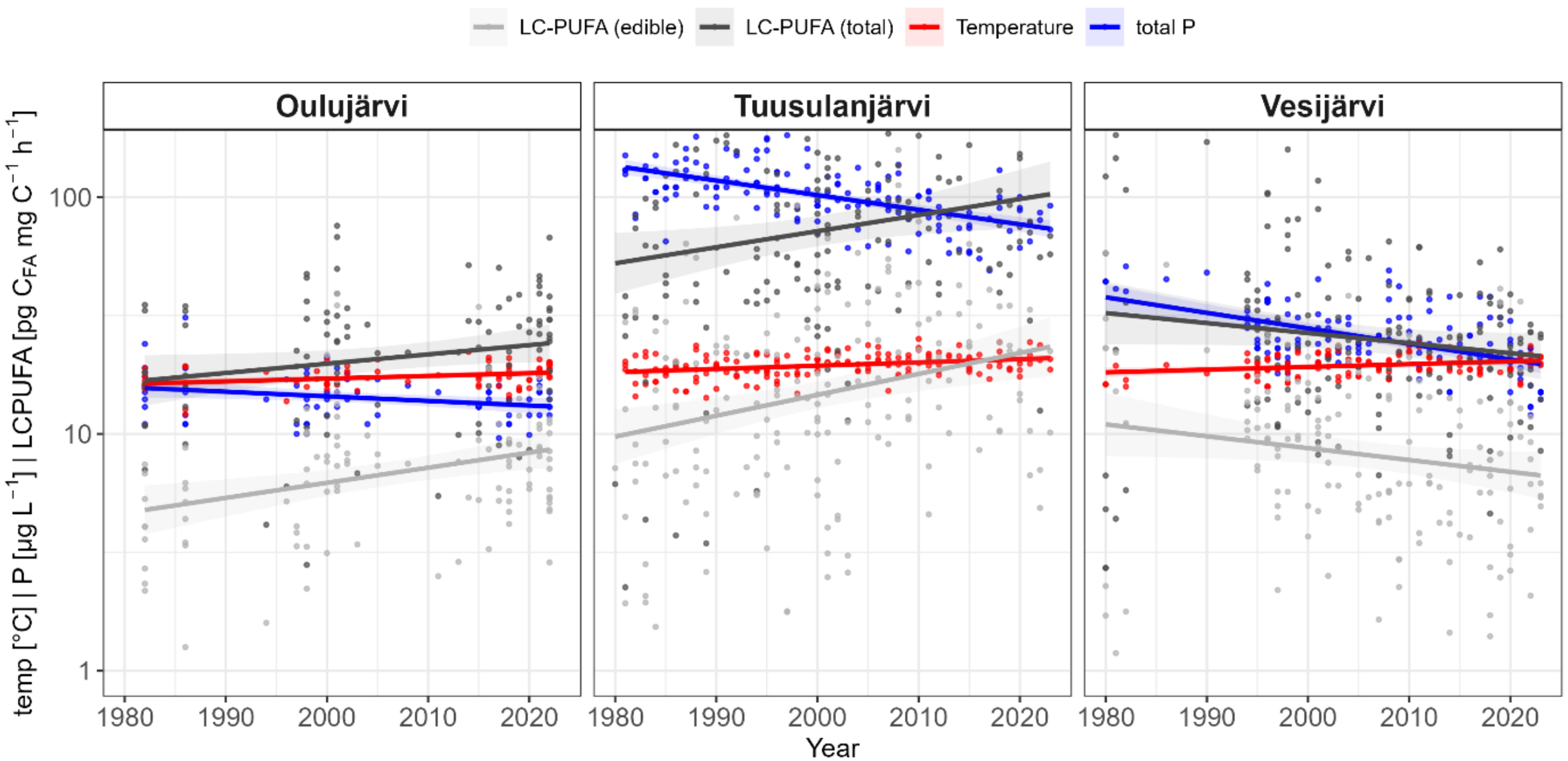

Figure S3. Long-term trends of three selected examples. In all lakes, a positive linear temperature trend of approx. + 0.5°C / 10 years, while significant negative trends in total phosphorus were observed, which, however, had different effects on each lake. In the case of the relatively cold, oligotrophic and northern Oulujärvi, the total biosynthesis of LC-PUFA increased, driven by the increase in water temperature. In the case of Tuusulanjärvi, LC-PUFA biosynthesis increased due to a significant decrease in total phosphorus, shifting the lake from eutrophic towards mesotrophic. In contrast, the total phosphorus concentration in Vesijärvi started near the optimum but decreased over the years, thereby reducing LC-PUFA biosynthesis.
